# Mammal Community Responses to Increasing Puma Activity in a Suburban Preserve

**DOI:** 10.1002/ece3.73775

**Published:** 2026-06-17

**Authors:** Chinmay Sonawane, Kevin Leempoel, Nicole Nova, Jordana M. Meyer, Trevor Hébert, Amelia Zuckerwise, Rodolfo Dirzo, Elizabeth A. Hadly

**Affiliations:** ^1^ Department of Biology Stanford University Stanford California USA; ^2^ Jasper Ridge Biological Preserve ('Ootchamin 'Ooyakma) Stanford University Stanford California USA; ^3^ Woods Institute for the Environment Stanford University Stanford California USA; ^4^ Department of Earth System Science Stanford University Stanford California USA

**Keywords:** behaviour, camera trapping, ecology of fear, mesopredator, predator, *Puma concolor*

## Abstract

Predators can shape ecosystems by directly reducing prey abundance and inducing fear‐driven changes in the behaviour of their prey and mesopredators, with potential cascading effects on lower trophic levels. However, these effects have been studied in landscapes with relatively low human presence. While some predator species coexist with people in human‐dominated landscapes, it remains unclear whether predators can fulfil their prey regulation functions in such environments. In a suburban preserve in the San Francisco Bay Area, USA, puma activity increased over nine years. We investigated if this increasing puma activity affected mesopredators (bobcats, coyotes and grey foxes), prey (black‐tailed deer and brush rabbits) and woody plants at a local scale using evidence from three analyses. First, convergent cross mapping analysis indicated that pumas influenced longitudinal changes in prey and mesopredator activity. Second, daily activity pattern analysis showed that prey and mesopredators decreased nocturnality, potentially to avoid pumas. Third, limited plant community sampling (three surveys during a 17‐year period) revealed that woody plant density increased as puma activity increased. Collectively, these analyses provided corroborating, preliminary signals that increasing puma activity coincided with changes in bobcats, coyotes and deer. However, inferences about any effects on foxes, rabbits and woody plants remain provisional and warrant stronger empirical confirmation. Nonetheless, as humans and predators increasingly overlap spatially, ensuring that large predators can persist and function ecologically in human‐dominated landscapes is a growing conservation priority.

## Graphical abstract

Using nine years of camera‐trap data from a suburban preserve, we found preliminary, convergent evidence that increasing puma activity coincided with changes in black‐tailed deer, bobcat and coyote activity. Evidence for changes in brush rabbit and grey fox activity, as well as woody plant density, is more tentative and warrants further study, but these results highlight the importance of evaluating the ecological roles of predators in human‐dominated landscapes.

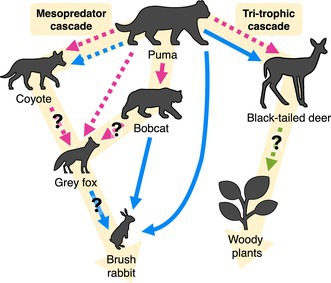

## 1 Introduction

Large predators structure ecosystems by affecting prey and mesopredators and, in turn, triggering indirect, cascading impacts on species throughout the food web (Terborgh and Estes 2013; Tossens et al. 2025). Two forms of trophic cascades are commonly recognised: mesopredator cascades, affecting subordinate competitors and their prey, and tri‐trophic cascades, affecting herbivorous prey and their food plants (Beschta and Ripple 2009; Prugh et al. 2009). Through these cascades, large predators regulate ecosystem functioning and provide services to ecological communities and people (Ripple et al. 2014). Initial research on trophic cascades demonstrated how the killing of prey and mesopredators can trigger cascading changes in species abundances, a density‐mediated effect (McLaren and Peterson 1994). However, the mere perception of predator presence may be more important than predation itself in triggering cascades (Zanette and Clinchy 2019). Known as “the ecology of fear”, predator presence can prompt prey and mesopredators to shift their space use and daily activity patterns, a behaviour‐mediated effect, with impacts on their food sources at lower trophic levels (Brown et al. 1999).

Much of the research on large predator effects has concentrated on ecosystems characterised by low human presence, even though 75% of the terrestrial surface on Earth has been significantly modified by humans (IPBES 2019). The extent to which large predators can still regulate ecosystems within human‐dominated landscapes remains unclear (Kuijper et al. 2016). Furthermore, in the U.S., small (< 5 km^2^) suburban and urban preserves constitute 25% of all terrestrial protected areas (UNEP‐WCMC and IUCN 2024; U.S. Census Bureau 2024). Despite their prevalence, these preserves are often dismissed for holding little ecological utility and incapable of providing value to wide‐ranging species (Schwartz and van Mantgem 1997). While such small preserves may not independently support large predators, they are increasingly incorporated by predators into their broader home ranges anchored in adjacent wilderness (Bateman and Fleming 2012). Therefore, in a world undergoing rapid anthropogenic change, there is a growing need to understand whether large predators can continue to impose community‐wide impacts in human‐dominated landscapes.

**FIGURE 1:**
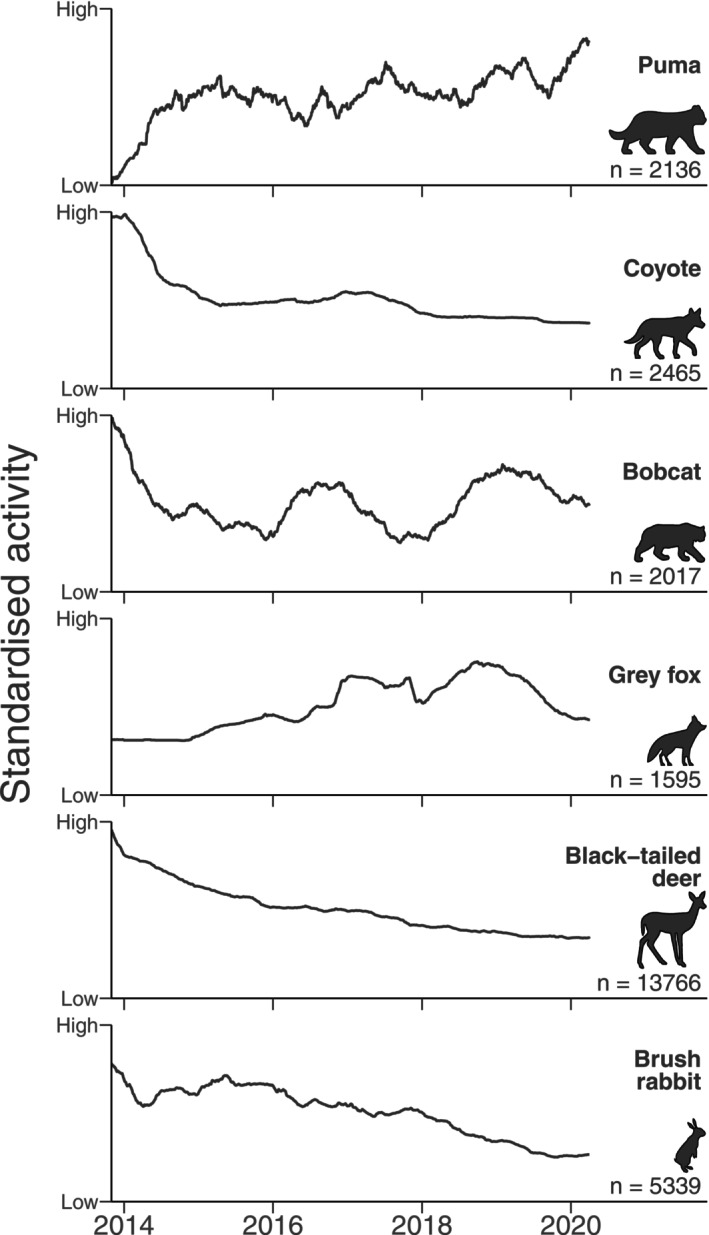
Change in activity of mammals at Jasper Ridge Biological Preserve ('Ootchamin 'Ooyakma). Time series of activity (standardised moving sum of independent detections per camera over a 365‐day window) of six mammal species from the 2012–2020 camera‐trap data (17 cameras). Low and high activity axis ticks approximate the lowest and highest standardised activity index values for each species and are included for visualisation purposes only. The total number of independent detections for each species is indicated by *n*.

We addressed this need by studying the ecological community at a suburban preserve in California, USA (Figure S1). Here, puma ( *Puma concolor* ) presence dramatically increased over time, enabling us to investigate the subsequent changes in mesopredators and prey (Figures 1 and S2) and the ensuing consequences for plant communities. We specifically studied the response of five mammalian species, including mesopredators (bobcat, *Lynx rufus* ; coyote, *Canis latrans* ; and grey fox, *Urocyon cinereoargenteus* , “fox”) and prey (black‐tailed deer, *Odocoileus hemionus* , “deer”; and brush rabbit, *Sylvilagus bachmani* , “rabbit”) due to their prevalence in our camera‐trap data (> 1500 independent detections for each species) and their well‐documented trophic (predation and herbivory) and non‐trophic (intraguild killing and resource competition) interactions with one another (Dyck et al. 2022; Farias et al. 2005; Karandikar et al. 2022; LaBarge et al. 2022; Meyer et al. 2020). Informed by these established top‐down interactions, we hypothesised two cascading chains of puma influence (Zanette and Clinchy 2019). First, we predicted that increased puma activity would be linked to deer reducing their use of the preserve—particularly at night when pumas were most active—resulting in increased woody plant density from reduced herbivory (a tri‐trophic cascade). Second, we predicted that bobcats and coyotes would also avoid the preserve during the period of high puma activity, particularly at night, thereby ecologically releasing foxes at night. Increased nocturnal fox activity, in turn, would suppress rabbit activity, especially at night, producing a mesopredator cascade. Our hypotheses were evaluated using three approaches, analysing if pumas: (1) contributed to longitudinal changes in animal activity over 6+ years, (2) influenced the daily activity patterns (i.e., diurnality) of other species and (3) indirectly affected plant communities. In these approaches, we also considered bottom‐up hypotheses in which changes in prey influenced pumas.

## 2 Methods

### 2.1 Study Area

We monitored wildlife within and outside of Jasper Ridge Biological Preserve ('Ootchamin 'Ooyakma) (California, USA), a 4.9 km^2^ suburban preserve situated in the San Francisco Bay Area and on the eastern foothills of the Santa Cruz Mountains. The preserve is partially fenced, allowing animals to move between the preserve and the Santa Cruz Mountains (Figure S1b). The preserve is characterised by a Mediterranean climate, with annual precipitation averaging 536 mm (range: 256–788 mm) and daily temperature averaging 14.5°C (95% of daily temperatures between 7.0°C–21.8°C) between 2014 and 2021 across chaparral, grassland and oak woodland ecosystems of the preserve (KCAWOODS19: Sun Field Station weather station).

Between 1972 and 2008, almost no signs of pumas were recorded in the preserve by previous reports, including a camera‐trap study from 2006 to 2008 (Dirzo et al. 2009). Between 2009 and 2012, pumas were recorded only on 54 days across 16 camera traps. After 2012, puma activity began to increase; however, the underlying drivers remain unclear. Cameras, active year‐round, began to annually record female pumas raising offspring, suggesting that the preserve acted as an area of refuge for raising offspring. This may have partly contributed to the markedly high year‐round puma activity in the preserve from 2015 onward (Figures 1, S2a and S3). This increase in puma activity provided the opportunity to assess mesopredators and prey responses, if any, through three approaches, outlined below.

### 2.2 Data Collection From Camera Traps

Between 2009 and 2018, 17 motion‐sensing cameras (BuckEye) were installed 40 cm above the ground, predominantly on fire roads and trails, rather than a grid‐based design, to maximise the detection of medium‐ and large‐sized mammals (Figure S1b, green and purple squares). The mean distance between the 17 cameras was 1303 m (range: 132–2751 m). Initially, volunteers identified the species in the captured images and these identifications were confirmed by at least one other volunteer. Beginning in December 2017, images were classified using a machine learning algorithm (Mathur and Khattar 2019). Because an individual animal may wander in front of the camera and trigger multiple photographs, we considered detections to be independent if two photographs of the same species at the same camera were separated by at least 30 min (Wang et al. 2015). Although we followed prior studies in selecting this 30‐min interval for simplicity, we acknowledge that it may introduce potential species‐specific biases, as differences in group size and movement rate may affect detection frequency.

### 2.3 Analysis of Longitudinal Changes in Animal Activity

We tested whether increasing puma activity over time influenced the activity indices of other animals, consistent with the ecology of fear. To do so, we created time series of each species' activity from the 17 preserve cameras (Figures 1 and S2). We subsequently identified potential relationships between these time series using a data‐driven method called convergent cross mapping (Sugihara et al. 2012). This method assumes that if species *X* influences species *Y* in a deterministic nonlinear dynamical system, then information about the time series for species *X* can be predicted from the time series of species *Y*. If the predicted time series of species *X* is similar to its observed time series, then convergent cross mapping deems species *X* to influence species *Y*. In doing so, convergent cross mapping distinguishes putative causality (*sensu* Granger 1969) from spurious correlation and indicates the direction of the association between two variables (i.e., bottom‐up or top‐down forcing).

To first create a time series of each species' activity, we summed the number of independent detections across the 17 cameras for each day for each species between 1 November 2012 and 31 March 2020. The number of independent detections per day was divided by the number of active cameras, as four of the 17 cameras were installed after 1 November 2012. Next, we calculated a right‐aligned moving sum over 365 days for each time series to minimise the effects of seasonality in the time series—strong cyclic patterns in the time series can compromise the efficacy of convergent cross mapping (Chang et al. 2017). Finally, we standardised each time series to zero mean and unit variance for unbiased comparability between the activity indices of each species (Sugihara et al. 2012; Nova et al. 2021). We also repeated these steps to create a time series using three spatially dispersed cameras (Figure S1b, purple squares) that were active from 1 November 2010 to 31 March 2020 (referred to as the “longer time series”). Due to the study area's size, we interpreted these time series as indices of activity (the “footprint” of each species, especially on trails) (Burton et al. 2015; Sollmann 2018). These changes in activity over time may be driven by: (a) more individuals using the area, (b) the same individuals using the area more frequently or (c) a combination of these two factors. However, without further information, our data could not identify whether species abundance or behaviour had changed.

We then used these time series (considered to be nonlinear, Figures S7 and S11) of activity indices to perform convergent cross mapping. Here, we provide a brief introduction to convergent cross mapping within the empirical dynamic modelling framework. These topics are covered in detail in Chang et al. (2017), Sugihara et al. (2012) and Tsonis et al. (2018). Empirical dynamic modelling begins with attractor reconstruction, which involves plotting the time series of each species' activity index in a multidimensional state space to recover the dynamics of the system. According to Takens' theorem, this attractor *A*, representing the dynamics of the system, can also be reconstructed using only one of the species' time series lagged against itself (e.g., *X*(*t*), *X*(*t*–τ), *X*(*t*–2τ) for variable *X* and time lag *τ*; *τ* = 1 in this study). This creates a univariate shadow attractor *A*_*X*_ that preserves the essential mathematical properties of the original attractor *A*. The optimal number of lagged times series (i.e., the embedding dimension, *E*) required for the shadow attractor was obtained by performing a nearest‐neighbour regression called simplex projection. The *E* that best predicted the time series (as measured by Pearson's correlation coefficient), was chosen for the reconstruction of the shadow attractor for each species (Figures S6 and S10).

Next, convergent cross mapping detects putative causal relationships between variables *X* and *Y* by comparing local points *X*(*t*) and *Y*(*t*) on shadow manifolds *A*_*X*_ and *A*_*Y*_, respectively. For example, if puma activity influences deer activity, then information about puma activity will be embedded in the dynamics of deer activity, such that the shadow attractor *A*_deer_ (constructed using the time series for deer activity) can reconstruct past values of puma activity. In other words, the shadow attractor constructed from the time series of deer activity is used to predict the time series of puma activity using simplex projection. If the predicted puma activity time series is similar to the observed puma activity time series, then convergent cross mapping suggests that pumas affect deer. The similarity between the predicted and observed puma activity time series is measured by Pearson's correlation coefficient (ρ), referred to as cross‐mapping skill, prediction skill or predictability. Greater predictability (ρ) implies a stronger influence of pumas over deer.

In an ideal model, predictability must increase and eventually plateau (converge) as more data (i.e., longer time series) are used in convergent cross mapping. This is because additional data in longer time series lead to more trajectories within the shadow attractor, and the denser shadow attractor improves the accuracy of prediction due to closer nearest neighbours. To evaluate whether the model converged as expected, we performed convergent cross mapping using different library lengths (each bootstrapped 1000 times), that were randomly subsampled from the complete time series. The 95% confidence interval (CI) of predictability was calculated using the 2.5th and 97.5th percentiles from the bootstraps. We then visually checked if predictability increased and plateaued with increasing library size (Figures S4, S5, S8 and S9).

Finally, to address the issue of confounding variables with strong periodicity (e.g., environmental, human and seasonal factors) influencing species at the preserve, we compared the empirical model (as described above) to a null model. For the null model, we created 1000 randomised surrogate time series of the putative driver species (i.e., the species influencing another) for convergent cross mapping, thereby losing any signal of causality, if present, in the observed time series. To account for spurious predictability based on neighbouring time‐dependence (i.e., serial correlation), we created surrogate time series using the Ebisuzaki method (Ebisuzaki 1997). This process preserved any periodic trends (including seasonal) and randomly shuffled the Fourier phases (the timing offsets of the signal's frequency components) of the observed time series. These surrogate time series were then used in lieu of the observed time series for the putative driver species for convergent cross mapping. We assessed if the predictability of this null model was smaller than that of the empirical model, when using the complete time series: this was evaluated with a one‐sided empirical *p* value ($=\frac{s+1}{1000+1}$ where s is the sum of replications when the predictability in the null model was greater than or equal to that in the empirical model). When *p* ≤ 0.05, the empirical model had greater predictability in at least 95% of the replications, indicating that the putative driver species, rather than confounding variables, significantly influenced another species. Convergent cross mapping was performed using the *rEDM* package in R (version 4.2.0) (Ye et al. 2018).

Unlike conventional correlation methods, convergent cross mapping first determines whether species *X* influences species *Y* using the process described above; this was then repeated for the converse interaction to see whether species *Y* influences species *X*. Convergent cross mapping can therefore differentiate between bottom‐up and top‐down effects between species pairs and identify which of these effects is stronger than the other.

### 2.4 Analysis of Changes in Daily Activity Patterns of Animals

We evaluated whether the presence of nocturnal puma activity coincided with cascading changes in the daily activity patterns of other animals. Unlike convergent cross mapping which analysed longitudinal changes in wildlife activity over years, this analysis examined changes to the diurnality and nocturnality of wildlife. Daily activity patterns were estimated using non‐parametric kernel density estimation of the timestamps of independent detections from the camera traps (Ridout and Linkie 2009). Timestamps were scaled by setting the sunrise and sunset times for each day as 0600 and 1800 h respectively to account for seasonal variation in light levels, which often regulate the daily activity patterns of terrestrial animals (Cermakian and Sassone‐Corsi 2002). Measures of nocturnality were calculated as the percentage of independent detections between 1800 and 0600 h.

Daily activity patterns of each species were compared under low and high puma activity. First, we compared activity patterns of each species during 1 November 2010 to 31 December 2012 (characterised by low puma activity) against that during 1 January 2015 to 31 March 2020 (characterised by high puma activity) inside the preserve. Second, we compared activity patterns of each species outside the preserve (characterised by low puma activity) against that inside the preserve (characterised by high puma activity) during 12 July 2019 to 31 December 2022. For this latter comparison across space, we installed four cameras (Browning; Figure S1b, blue squares) just outside of, but contiguous with, the preserve boundary. This triangular area (Figure S1b, in blue) was characterised by the same oak woodland/grassland mosaic as the preserve, though it was generally more open. Puma activity was low here, as the area was bounded by highly travelled roads (Sand Hill Road and Interstate 280) and the Stanford Linear Accelerator, a 3.2‐km physical barrier. This comparison included dates over the COVID‐19 pandemic, when human activity varied around the preserve, to ensure sufficiently large sample sizes for statistical analyses. Given these contextual differences and the limited number of cameras outside the preserve, this spatial comparison was intended as an anecdotal check for the temporal comparison, rather than a standalone, robust analysis.

Both these comparisons were evaluated by two metrics. First, we used the non‐parametric Watson's *U* ^*2*^ test, which tested for differences between two circular distributions. This test indicated if any changes in a species' daily activity patterns (from low to high puma activity) could be attributed to chance. Second, a non‐parametric estimator of overlap coefficients quantified the intersecting area under the two density curves of the daily activity patterns of each species. This coefficient estimated the degree of similarity between daily activity patterns of a given species under low and high puma activity. As recommended by Ridout and Linkie (2009), we used the ∆_4_ estimator for the overlap coefficient, unless the smaller sample had fewer than 75 independent detections, in which case, we used the ∆_1_ estimator. The 95% CIs for these overlap coefficients were estimated with 1000 bootstraps. This coefficient also measured the overlap between the activities of two species. For simplicity, statistically significant changes in these percentages were estimated by comparing the overlap between two 95% CIs. We note that this approach conservatively detects statistically significant changes, as overlapping 95% CIs may still be statistically significantly different (Austin and Hux 2002). Analyses were performed in R using the *circular*, *overlap* and *suncalc* packages (Agostinelli and Lund 2022; Ridout and Linkie 2009; Thieurmel and Elmarhraoui 2019).

Our interpretation of changes in daily activity patterns followed that of other studies (e.g., Vallejo‐Vargas et al. 2022). If top‐down effects regulated daily activity patterns, then species would adjust their activity to avoid predation and competition. This would be reflected by a: (i) significant change in the activity pattern of a given species (from low to high puma activity) using the Watson's *U* ^*2*^ test and (ii) decrease in the overlap coefficient of the given species and its predator. Conversely, if bottom‐up effects regulated activity patterns, then species would adjust their activity to increase encounters with prey. This would be reflected by: (i) a significant change in the activity pattern of a given species (from low to high puma activity) using the Watson's *U* ^*2*^ test and (ii) an increase in the overlap coefficient of the given species and its prey. However, these indicators provide only inferential support and do not by themselves confirm top‐down or bottom‐up regulation, because similar shifts in daily activity patterns can arise from unmeasured environmental variation, changes in density or other behavioural processes.

### 2.5 Analysis of Changes to Woody Plant Density

To assess the changes to vegetation, we sampled woody plant density and plant cover at 12–14 quadrats around the preserve, interspersed among the cameras, in the spring of 2006, 2015 and 2023 (Figure S1b, white circles). In 2006, quadrat locations were selected using a hexagonal grid design to separate the preserve into 12 equal‐sized hexagons; 10 × 10 m quadrats were then marked at the centre of each hexagon. To sample woody plants, we counted and measured the basal diameters of all woody plants with a basal diameter of ≥ 1 cm that were within the quadrats. Differences in woody plant density between years were evaluated using Mann–Whitney *U* tests, with Holm‐Bonferroni corrections applied to *p* values (exact *p* values could not be calculated due to ties).

To sample plant cover, we used a line intercept method, whereby transect tapes were laid out vertically and horizontally at each one metre mark in the 10 × 10 m quadrat. At each intercept, we noted the absence or presence of plant cover at ground or shin‐height. Differences in plant cover between years were evaluated using Chi‐squared tests with Yates's continuity corrections, and with Holm‐Bonferroni corrections applied to *p* values.

We repeated woody plant density and plant cover sampling in the quadrats in 2015 and 2023. In these years, some of the original quadrats were too densely vegetated to allow for sampling; thus, nearby areas within the same vegetation communities were sampled. This likely implies that we underestimated woody plant density. In spring of 2023, area closure due to nesting golden eagles also prevented the sampling of another original quadrat.

## 3 Results

### 3.1 Longitudinal Changes in Species Activity

**FIGURE 2:**
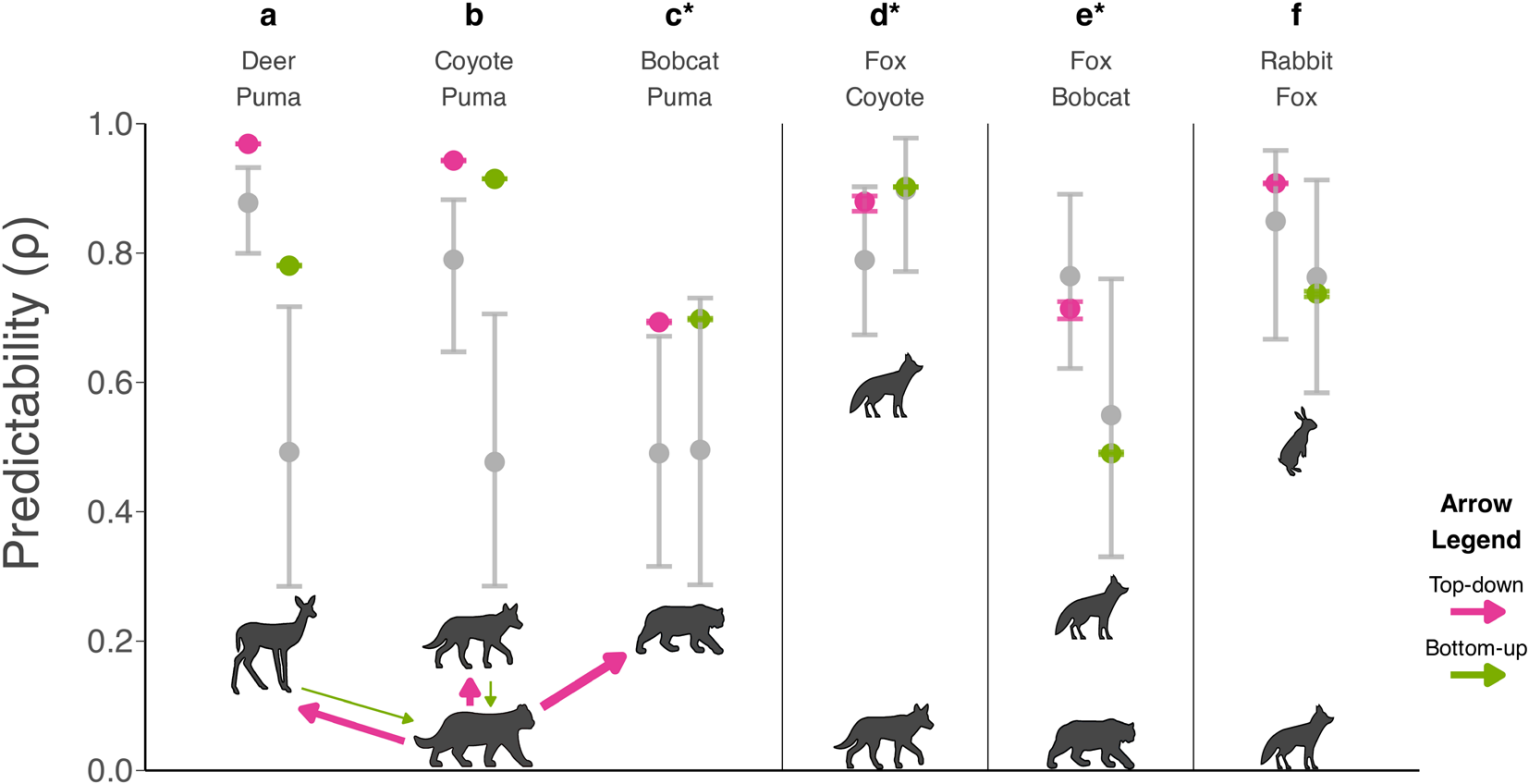
Hypothesised trophic cascade interactions assessed by convergent cross mapping using the shorter time series. Dots indicate mean predictability (ρ) from 1000 bootstraps, with error bars capturing the 95% CIs when using the complete time series (length of time series = 2326 days). Pink dots assessed if activity of bottom species influenced activity of top species (top‐down interaction), and green dots assessed if activity of top species influenced activity of bottom species (bottom‐up interaction). Grey dots indicate predictabilities from the respective null models. Arrows between species indicate significant interactions (predictability of empirical model > predictability of null model). In bidirectional interactions, the interaction with higher predictability is indicated by a larger arrow. Asterisks on panel labels indicate that significant top‐down interactions were detected by convergent cross mapping in the three‐camera‐trap dataset (Figure S8).

Out of 24 hypothetical interactions (12 pairs of animal species) that were analysed, convergent cross mapping detected three top‐down, three bottom‐up and one competitive interaction when using data from the 17 cameras between 2012 and 2020 (Figure 2, Figure S4 and Table S1). Analysis of the longer time series yielded additional interactions (Figure S8 and Table S2). When interactions were bidirectional (species *X* was significantly influencing species *Y*, and species *Y* was significantly influencing species *X*, relative to their respective null models), the interaction with the higher predictability (according to the 95% CI, though see Austin and Hux 2002) was considered stronger. This eliminated two significant bottom‐up interactions, as two species pairs exhibited bidirectional interactions (Figure 2a,b).

Convergent cross mapping revealed that pumas directly influenced the activity of four other species. Pumas and deer bidirectionally influenced one another, with the top‐down effect more pronounced than the bottom‐up effect in the shorter time series (Figure 2a) and equal in strength in the longer time series (Figure S8d). A similar bidirectional interaction pattern was observed between pumas and coyotes (Figure 2b and Figure S8a). Puma activity also influenced bobcats (Figure 2c and Figure S8b). In the longer time series, bobcats, coyotes and pumas exerted significant influence on foxes (Figure S8c,h,j). Coyotes had top‐down effects on rabbits in the longer time series (Figure S8i), while the reverse was observed in the shorter time series (Figure S4i). Rabbits also influenced deer in the shorter time series (Figure S4f), and bidirectionally influenced pumas in the longer time series, with effects equal in both directions (Figure S8e).

### 3.2 Changes to Daily Activity Patterns of Species

**FIGURE 3:**
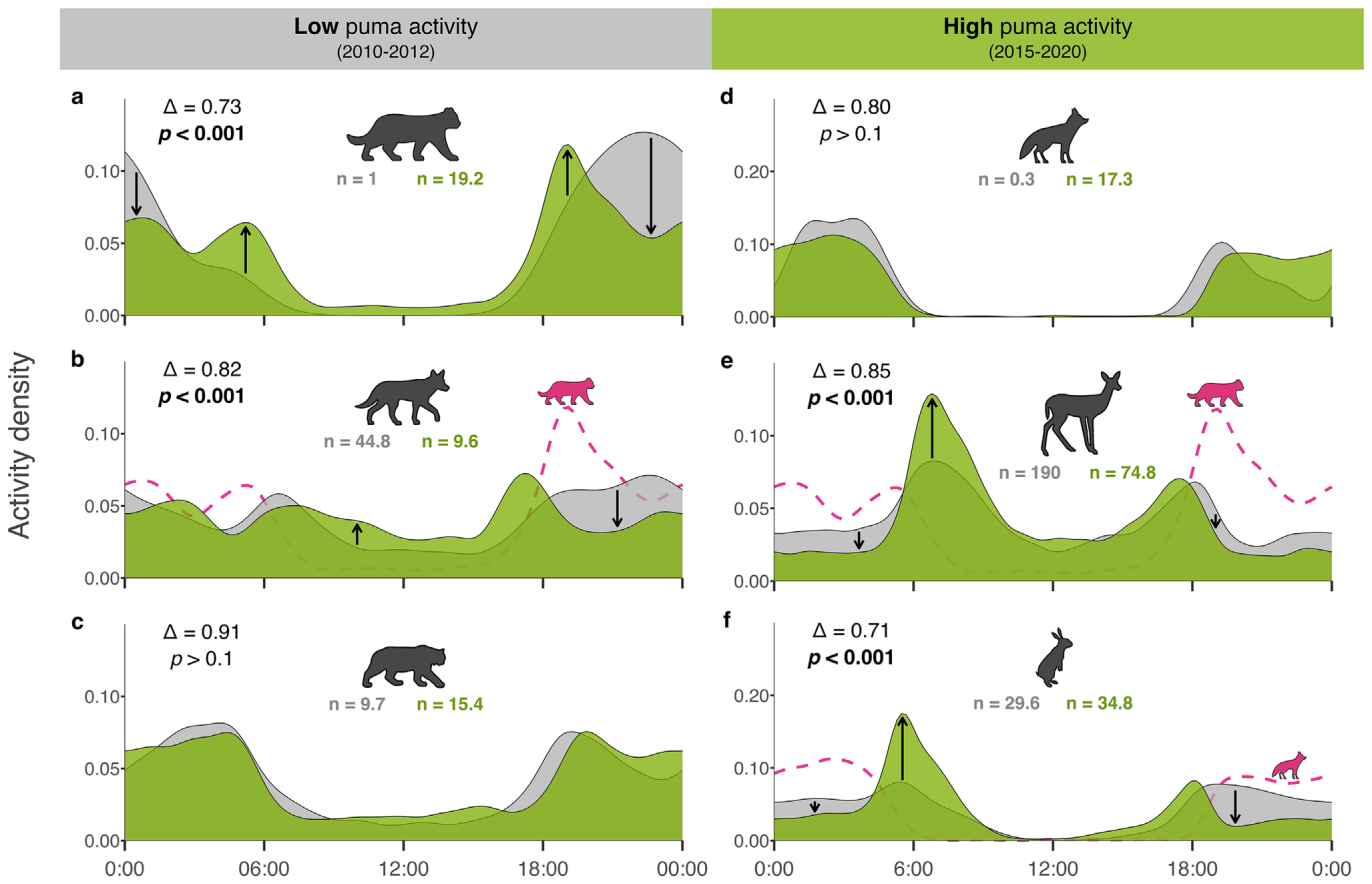
Kernel density estimation of daily activity patterns under low and high puma activity. (a–f) Activity patterns of species during 2010–2012 (grey) and 2015–2020 (green) at Jasper Ridge Biological Preserve ('Ootchamin 'Ooyakma) (low and high puma activity across time). Overlap coefficient (∆) indicates similarity between activity patterns during low and high puma activity for each species (95% CIs in Table S3). *p* value indicates if the two activity patterns were different using the Watson's *U* ^*2*^ test (test statistics in Table S3). Where activity patterns are different, arrows indicate how activity patterns changed from low to high puma activity. The number of independent detections per year per camera for each sample is indicated by n. Activity patterns, under high puma activity, of species that pose predation and intraguild killing threats are displayed in dashed lines.

Our analysis found that coyotes, deer, foxes and rabbits changed their daily activity patterns as puma activity increased, while bobcats exhibited no changes. Approximately 85% of puma activity was recorded at night in the preserve, though peak activity shifted from nocturnal hours to sunset during 2015–2020 (Figure 3a). During years of high puma activity in the preserve (2015–2020), coyotes and deer reduced their nocturnal activity by 25% and 34%, respectively (Figure 3b,e) compared to the period of low puma activity (2011–2012). Without these behavioural changes, coyotes and deer would have experienced 16% (95% CI: 9%–23%) and 24% (95% CI: 18%–29%) greater overlaps with the activity patterns of pumas, respectively (Table S5). When considering the daily activity patterns in the area outside the preserve, with low puma activity, corroborating changes were recorded for deer (Figure S12e, Table S6). Coyote activity also dropped during peak puma activity hours (after sunset) when comparing daily activity patterns inside and outside the preserve. However, overall nocturnality and overlap with pumas did not change considerably (Figure S12b, Table S6).

Additionally, changes in the activity patterns of even smaller species were detected. While foxes remained consistently nocturnal, they were more active earlier in the night inside the preserve, relative to outside (Figure S12d). This shift increased their overlap with bobcats and coyotes (Table S6). However, these changes were not observed when comparing fox activity within the preserve between 2010–2012 and 2015–2020 (Figure 3d and Table S5). Rabbits were originally nocturnal in the preserve, and this nocturnality decreased by 24% under years of high puma activity (Figure 3f). After these activity changes, the overlap between rabbits and foxes, their main predator, was reduced by 41% (95% CI: 36%–46%) (Table S5). Similar changes occurred for rabbits when comparing their activity patterns inside and outside the preserve (Figure S12f and Table S6).

Beyond these top‐down interactions, pumas increased their overlap with deer, among other species, from 2010–2012 to 2015–2020 in the preserve (Table S5). However, these bottom‐up effects were not replicated in the comparison outside the preserve (Table S6).

### 3.3 Changes to Woody Plant Density

**FIGURE 4:**
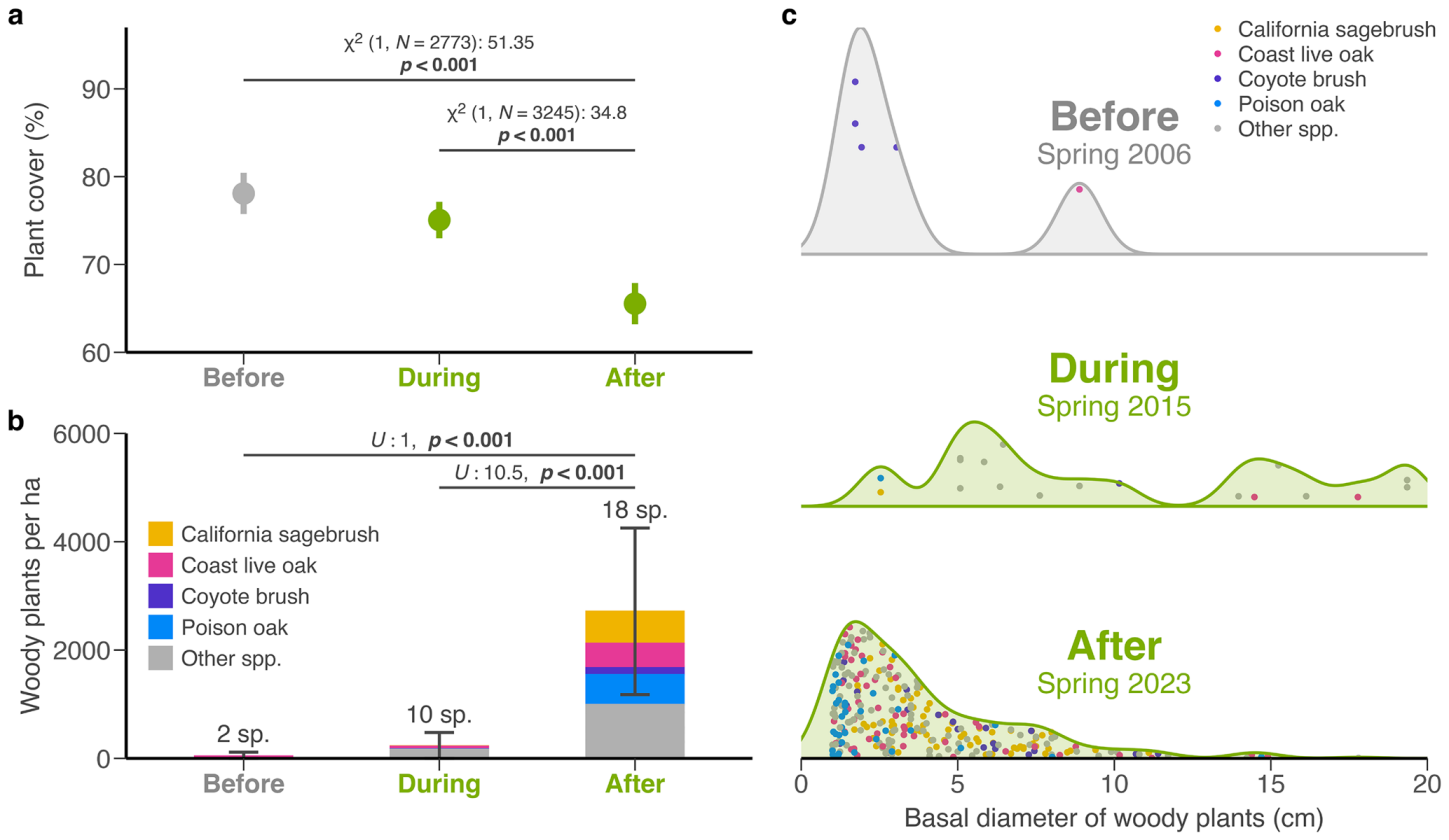
Vegetation composition before, during and after puma activity increases. (a) Plant cover estimated from 10 × 10 m quadrats, using the line intercept method. Statistically significant differences between years were evaluated using Chi‐squared tests. (b) Woody plant density estimated by counting woody plants with basal diameter ≥ 1 cm in each of the sampled 10 × 10 m quadrats. Statistically significant differences between years were evaluated using Mann–Whitney *U* tests. In a–b, error bars indicate 95% CIs and Holm–Bonferroni corrections are applied to *p* values. (c) Distribution of basal diameters of woody plants with basal diameters ≥ 1 cm and ≤ 20 cm. In a–c, grey indicates period with low puma activity (2006) and green indicates periods with high puma activity (2015 and 2023).

Between 2006, 2015 and 2023, plant cover and woody plant density inside the preserve significantly changed (Figure 4). Plant cover decreased by 16% (95% CI: 10%–21%) over the 17 years (Chi‐squared test: *χ* ^2^(1, *N* = 2773) = 51.35, Holm–Bonferroni corrected *p* < 0.001). Meanwhile, woody plant density increased by 64‐fold, primarily driven by the presence of smaller plants (Mann–Whitney *U* test: *U* = 1, Holm–Bonferroni corrected *p* < 0.001). Species richness of woody plants found within the sampled quadrats also increased from two to 18 species over 17 years. Among these plants, three key plants found in deer diets (California sagebrush, *Artemisia californica* ; coast live oak, *Quercus agrifolia* ; and poison oak, *Toxicodendron pubescens* ) (Meyer et al. 2020) exhibited the highest densities in 2023. Importantly, these results remained robust even when analysing only the eight quadrats that were sampled in both 2006 and 2023 (Figure S14).

## 4 Discussion

**FIGURE 5:**
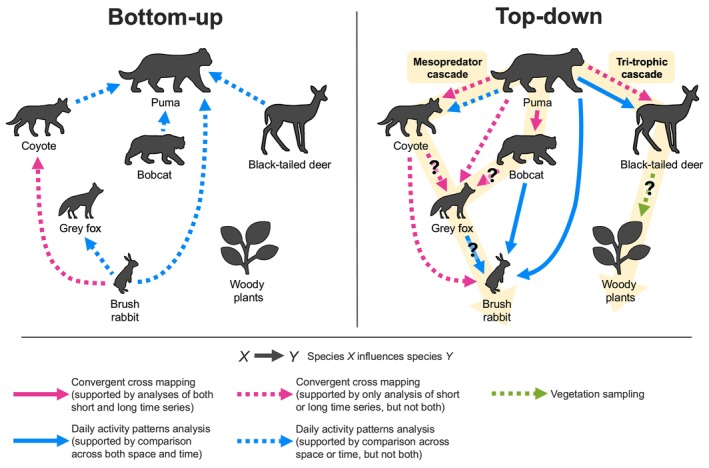
Summary of bottom‐up and top‐down interactions recorded at Jasper Ridge Biological Preserve ('Ootchamin 'Ooyakma). Line types indicate the analyses that substantiate the interactions. Competitive interactions and bidirectional interactions where both opposing interactions are of similar strength are not shown. The hypothesised mesopredator and tri‐trophic cascade pathways are highlighted in yellow. Question marks indicate tentative interactions within the hypothesised cascades that warrant further study.

Our study highlights the potential for top predators to influence community structure, while also emphasising the complexity of detecting cascading effects across trophic levels. The results provide preliminary support for puma‐associated changes in deer and coyote activity across both convergent cross mapping and daily activity pattern analyses, whereas evidence for changes in bobcats is limited to the convergent cross mapping results (Figure 5). While the results also indicate changes in foxes, rabbits and woody plants, these cascading impacts fall short of the evidentiary standard needed to infer mesopredator and tri‐trophic cascades (Ford and Goheen 2015).

As pumas increased their activity in the preserve, their primary prey, deer, exhibited reduced activity and temporal segregation—potentially to avoid predation—consistent with similar systems elsewhere in California (Gaynor et al. 2022; Patten et al. 2019). Consequently, this decline in overall deer activity may have reduced herbivory on woody plants, although this association remains tentative (Figure S15). An ongoing exclosure experiment at the preserve provides experimental evidence that deer herbivory can significantly suppress the woody plant survival, though this does not conclusively demonstrate that herbivory caused vegetation changes across the preserve (Figure S16). The observed increases in woody plant density were also associated with decreasing overall plant cover, potentially reflecting suppression of herbaceous plants on the ground by the proliferation of woody plants (House et al. 2003). Furthermore, the observed inter‐annual changes in vegetation at Jasper Ridge Biological Preserve ('Ootchamin 'Ooyakma) appear unrelated to variations in rainfall and temperature across the sampled years (Figures S17, S18 and Table S7). Nevertheless, our data only provided three snapshots of vegetation composition across the 17‐year span. As such, vegetation changes could reflect unmeasured ecological drivers (e.g., atmospheric CO_2_, episodic recruitment years, fog inputs, herbaceous competition, soil moisture and nitrogen availability) or random sampling variation, rather than trophic interactions. Therefore, while these puma‐deer‐woody plant changes are consistent with a potential tri‐trophic cascade, conclusive evidence will require additional vegetation sampling and analyses that explicitly evaluate alternative explanations, including the role of abiotic drivers and small mammals.

Similarly, the hypothesised mesopredator cascade was also poorly substantiated, especially at the lower trophic levels. The activity of bobcats and coyotes changed as puma activity increased, potentially to avoid competition and predation (Koehler and Hornocker 1991; Meyer et al. 2020). Coyotes also temporally avoided pumas, corroborating findings from the Santa Cruz Mountains (Wang et al. 2015, 2020). Foxes appeared to have benefitted from the reduced activity of other mesopredators: under high puma activity, foxes occupied the spatial (the preserve) and temporal (nocturnal) niches previously filled by coyotes, consistent with findings from Chamberlain and Leopold (2005), Egan et al. (2021) and Wang et al. (2015). This increase in fox activity, particularly at night, corresponded with their prey, rabbits, shifting their activity to morning hours, likely to reduce predation risk (Elbroch and Allen 2013; Meyer et al. 2020). Additively, bobcats may have also directly driven this change in rabbits (Tables S5 and S6), as rabbits constitute an important diet item for bobcats too (Meyer et al. 2020). However, these results should be interpreted cautiously, as unmeasured factors could also explain these observed changes in fox and rabbit activity. Overall, puma activity coincided with shifts in bobcat and coyote activity, but evidence connecting these changes to fox and rabbit responses is weaker and remains unresolved.

In the absence of local population trends on the focal species, we cautiously draw on the following regional trends for ecological context, acknowledging their speculative application to our study area and period. Puma populations are stable in the Santa Cruz Mountains (Nisi et al. 2023), as are bobcat populations across California more broadly (Roberts and Crimmins 2010), though unquantified at the local level. Similarly, across the study period, deer and rabbit populations may be stable or increasing in California, despite historic declines (Mule Deer Working Group Technical Committee 2025; Furnas et al. 2020; Brown et al. 2018). Coyote populations may be increasing based on sightings and conflict reports across the Bay Area, though these may not accurately reflect abundance trends (Wilkinson et al. 2023; Furnas et al. 2020). Limited information is available on fox populations (Allen et al. 2021), with numbers susceptible to periodic crashes from canine distemper outbreaks (Ryan and Leikam 2022). Due to their limited specificity to our study area and period, these trends should be interpreted with caution. Indeed, future research that uses spatially explicit capture‐recapture models to estimate abundance could better evaluate the responses of mammals and distinguish whether the observed results here were driven primarily by behaviour‐mediated or density‐mediated pathways. Similarly, the woody plant changes observed from limited sampling raise the possibility of indirect puma‐associated effects on vegetation and should be evaluated with more frequent and spatially extensive surveys.

A necessary condition for predator influence is top‐down forcing (Ripple et al. 2016). While bottom‐up effects were present at Jasper Ridge Biological Preserve ('Ootchamin 'Ooyakma), top‐down effects were generally more common and stronger than bottom‐up effects. Among the six detected bottom‐up effects, three involved species pairs that exhibited bidirectional interactions in convergent cross mapping. In the puma‐coyote and puma‐deer species pairs, the top‐down effect was stronger in the shorter time series, while the two directions were of comparable strength in the longer series. These bidirectional interactions, when detected by convergent cross mapping, may be indicative of strong unidirectional interactions, in which a tight coupling of the two species results in synchronised activity indices (Ye et al. 2015). Therefore, strong top‐down effects from pumas on coyotes and deer may influence tight coupling among these species pairs, leading convergent cross mapping to detect bidirectionality. A third bottom‐up effect, from rabbits to pumas in the longer time series, was similarly close in strength to the top‐down effect, leaving the dominant interaction's direction unresolved. We acknowledge that in the analysis of the shorter time series, rabbits influenced coyote activity, suggesting that coyotes experienced bottom‐up effects in 2012–2020. Similarly, shifts in fox and puma daily activity patterns increased their overlap with their respective prey. Nonetheless, while bottom‐up effects were detected at the preserve, these were largely overshadowed by top‐down effects during the study period (Figure 5). 

While convergent cross mapping can discern causality (*sensu* Granger 1969) from spurious correlation (Tsonis et al. 2018), making it unlikely that other variables confound the species interactions detected here, future research should still quantify the contributions of these variables to the spatiotemporal dynamics of animals at the preserve (Ford and Goheen 2015). Human activity, for instance, is well‐known to influence wildlife, though its impact at the preserve appears limited upon initial examination. Convergent cross mapping did not detect effects of human activity on wildlife (Figures S5 and S9). Outside the preserve, traffic increased over the study period on the nearest monitored road (intersection of California State Route 84 and Portola Rd., approximately 1 km from the preserve) (State of California 2022), likely increasing daytime noise pollution. In contrast, the human population in neighbouring communities of Portola Valley and Woodside grew by just 1% from 2010 to 2020 and light pollution changed minimally (Lorenz 2022; U.S. Census Bureau 2021). Interestingly, species such as coyote, deer, puma and rabbit reduced their nocturnality, and increased their activity overlap with people over time (Figure S13 and Table S5), counter to expectations of how human disturbance affects animals (Gaynor et al. 2018). Even as the degree of human activity differed from inside to outside of the preserve, the changes in daily activity patterns of deer and rabbits in the spatial comparison remained consistent with the temporal comparison (Tables S3 and S4). Furthermore, the COVID‐19 lockdown, which limited human activity at the preserve, had minimal effects on wildlife diurnality (Béllo Carvalho et al. 2026). While these analyses cast further doubt on the role of people independently driving the observed changes in animals in this controlled‐access preserve, human activity may have provided prey species with a potential refuge from predators (Gaynor et al. 2025). Future research could explicitly test this human shield hypothesis by quantifying predator–prey temporal overlap as a function of human activity or proximity to infrastructure (e.g., the field station) at the preserve.

Similarly, if environmental factors, such as California's increasing heat and drought conditions, were primarily driving the observed spatiotemporal dynamics, then animals could be expected to have shifted towards nocturnality when ambient temperatures were lower to minimise energy expenditure and water loss (Davimes et al. 2017). Instead, these species shifted away from nocturnality. It is possible that these other factors may have had secondary effects on animals. For example, environmental factors and human disturbance may explain why many species switched to crepuscular activity, rather than diurnal activity. However, our quantitative analyses here provide preliminary evidence that increasing puma activity was a major contributor to the changes in animal activity at the preserve.

Importantly, our study area is small relative to the home ranges of pumas in the Santa Cruz Mountains, which vary from 20 to 170 km^2^ (Nickel et al. 2021). Therefore, changes in puma demographics or movement may contribute to the observed changes in puma activity in Figure 1. However, this is less likely for bobcats, coyotes and deer, whose home range sizes of 3, 5 and 1–11 km^2^, respectively, in similar Californian sites, are comparable to the size of the study area (Kie et al. 2002; Riley et al. 2003). An alternative hypothesis may suggest that these prey and subordinate competitors of pumas changed their movements or experienced demographic shifts independently of top‐down forcing from pumas. This implies that bobcats, coyotes and deer simultaneously left the preserve for less‐suitable habitat in the adjacent residential areas, which is unlikely without a strong forcing mechanism. While further research is required to conclusively rule out alternative hypotheses, our convergent cross mapping results suggest that the observed dynamics are unlikely to be explained solely by an unmeasured external factor acting simultaneously on these species (e.g., the Moran effect) (Sugihara et al. 2012). Finally, despite the spatial limitations of this study, we highlight the relevance of our findings to the 82% of U.S. protected areas that are smaller than 5 km^2^ (UNEP‐WCMC and IUCN 2024). Given that a third of these are bordering or within urban areas (U.S. Census Bureau 2024), we emphasise that these small protected areas, in areas of landscape connectivity, may preserve the ecological functions of even wide‐ranging species in the absence of heavy human activity.

Further, our analyses benefitted from nine years of continuous camera‐trap monitoring at Jasper Ridge Biological Preserve ('Ootchamin 'Ooyakma). Globally, 99% of camera‐trap surveys do not monitor beyond a 5.4‐year period (median study period = 8 months; Burton et al. 2015). Despite using fewer cameras than most studies (median = 31 cameras or 0.27 cameras/km^2^; this study = 17 cameras or 3.47 cameras/km^2^) due to the smaller size of our study area, our camera‐trapping effort (measured by trap days) was still greater than all 174 camera‐trap studies reviewed by Burton et al. (2015). Our 38,000+ independent detections (excluding humans, total detections across all analyses), captured over 61,000+ trap days, compensated for our reliance on trail‐based camera traps, which can contribute to biases in species detection. When datasets contain 1400 or more camera‐trap days, inferences of community dynamics are likely unaffected by non‐random camera‐trap placement (Cusack et al. 2015). However, because our cameras were primarily placed on trails, inferences about species interactions may apply mainly to trail use rather than the preserve as a whole. Deer and mesopredators could have reduced apparent overlap with pumas by shifting activity off‐trail. Therefore, our data provide valuable insights into interactions between medium‐ and large‐sized animals on trails; however, whether off‐trail areas function as spatial refuges within the preserve warrants closer examination.

Due to the study area's proximity to densely populated areas, highways and agricultural lands, the impacts of pumas may influence people as well (Figure S1). Interstate 280, a major Bay Area highway, sits approximately 1 km north of the preserve and has the highest rate of animal‐vehicle collisions in California, annually costing 117,000 USD per kilometre of highway in medical expenses and property damage in 2021 (Figure S19; Shilling et al. 2021). If the puma‐induced changes in deer around nearby roads are similar to the changes inside the preserve, then reductions in deer activity at dusk (when deer‐vehicle collisions peak) and overall deer activity may result in fewer deer‐vehicle accidents, thereby saving lives and minimising property damage (Gilbert et al. 2017; Huijser and Begley 2009; Raynor et al. 2021). However, the opposite outcome is also possible: if deer reduce use of the preserve and shift movement into surrounding residential and highway areas, collisions could increase. Quantifying such outcomes and resolving these competing hypotheses may help inform coexistence strategies, particularly as explosive urbanisation threatens pumas in California (Burdett et al. 2010). Overall, our results motivate further study of how predators can have far‐reaching implications for not only wildlife but also people.

## Author Contributions

**Chinmay Sonawane:** formal analysis (equal), investigation (supporting), methodology (equal), writing – original draft (equal), writing – review and editing (equal). **Kevin Leempoel:** formal analysis (equal), investigation (supporting), methodology (equal), writing – original draft (equal), writing – review and editing (equal). **Nicole Nova:** formal analysis (equal), methodology (equal), writing – original draft (equal), writing – review and editing (equal). **Jordana M. Meyer:** methodology (supporting), writing – original draft (supporting), writing – review and editing (equal). **Trevor Hébert:** investigation (lead), methodology (equal), software (lead), supervision (supporting), writing – review and editing (equal). **Amelia Zuckerwise:** investigation (supporting), methodology (supporting), writing – review and editing (equal). **Rodolfo Dirzo:** investigation (supporting), methodology (supporting), supervision (supporting), writing – review and editing (equal). **Elizabeth A. Hadly:** conceptualization (lead), methodology (equal), supervision (lead), writing – review and editing (equal).

## Funding

This work was supported by Schweizerischer Nationalfonds zur Förderung der Wissenschaftlichen Forschung (Grant P2ELP3_175075), Quad Fellowship, Philanthropic Educational Organization, National Science Foundation (Grants 0430448 and 0934210), Howard Hughes Medical Institute and Stanford University.

## Conflicts of Interest

The authors declare no conflicts of interest.

## Supporting information


**Figure S1:** Study area. (a) Location of Jasper Ridge Biological Preserve ('Ootchamin 'Ooyakma) in the Bay Area, California, USA. Purple polygon indicates the area of the preserve. The preserve sits in between the suburbia of the Bay Area and the Santa Cruz Mountains. Areas highlighted in pink indicate presence of human populations (Meta 2020). (b) Locations of the cameras and vegetation quadrats inside and outside the preserve. Colours in image have been enhanced to better delineate oak woodland and grassland habitats. Note building structures around preserve, indicating presence of people.
**Figure S2:** Change in activity of mammals at Jasper Ridge Biological Preserve ('Ootchamin 'Ooyakma) from the camera‐trap datasets. Time series of activity (standardised moving sum of independent detections per camera over a 365‐day window) from three cameras in November 2010 to March 2020 (“long time series”, in green) and from 17 cameras in November 2012 to March 2020 (“short time series”, in black). Respective n values indicate the total number of independent detections for each species for each of the two datasets.
**Figure S3:** Puma kittens at Jasper Ridge Biological Preserve ('Ootchamin 'Ooyakma) in 2013 (a), 2015 (b), 2016 (c, d) and 2018 (e).
**Figure S4:** Wildlife interactions detected by convergent cross mapping using the seventeen‐camera‐trap dataset from 2012 to 2020. Shaded areas indicate 95% CIs of predictability (ρ), using the 2.5th and 97.5th percentiles from the 1000 bootstraps. Pink shading indicates if activity of species on the left influenced that on the right, and green shading indicates if activity of species on the right influenced that on the left. Blue and grey shading indicate results from the respective null models. Pink arrow indicates left species significantly influenced right species (predictability of empirical model > predictability of null model), while green arrow indicates right species significantly influenced left species. In bidirectional interactions, the interaction with higher predictability is indicated by a larger arrow.
**Figure S5:** Human‐wildlife interactions detected by convergent cross mapping using the seventeen‐camera‐trap dataset from 2012 to 2020. Shaded areas indicate 95% CIs of predictability (ρ), using the 2.5th and 97.5th percentiles from the 1000 bootstraps. Pink shading indicates if activity of humans influenced activity of animal, and green shading indicates if activity of animal influenced activity of humans. Blue and grey shading indicate results from the respective null models. No interactions were detected.
**Figure S6:** Optimal embedding dimension for the seventeen‐camera‐trap dataset from 2012 to 2020. Simplex projection determined the optimal embedding dimension that best “unfolded” the dynamics, as measured by predictability (ρ). Optimal embedding dimension for each species, that was used for convergent cross mapping, is marked by a pink dashed line.
**Figure S7:** Evidence of nonlinear dynamics for the seventeen‐camera‐trap dataset from 2012 to 2020. Sequential locally weighted global linear map (S‐map) forecasting method fitted local linear maps, with the localisation parameter (*θ*) assigning weights to points depending on their location in relation to the point to be predicted. When *θ* = 0, all points are weighted equally and the S‐map model corresponds to an autoregressive model (i.e., a linear model). When *θ* > 0, nearby points receive greater weights and the S‐map corresponds to a nonlinear model. For bobcats, coyotes, deer, foxes, humans and rabbits, predictability (ρ) increased when *θ* > 0, thereby indicating nonlinear dynamics. At higher values of *θ*, predictability degrades as the S‐map model overfits.
**Figure S8:** Wildlife interactions detected by convergent cross mapping using the three‐camera‐trap dataset from 2010 to 2020. Shaded areas indicate 95% CIs of predictability (ρ), using the 2.5th and 97.5th percentiles from the 1000 bootstraps. Pink shading indicates if activity of species on the left influenced that on the right, and green shading indicates if activity of species on the right influenced that on the left. Blue and grey shading indicate results from the respective null models. Pink arrow indicates left species significantly influenced right species (predictability of empirical model > predictability of null model), while green arrow indicates right species significantly influenced left species. In bidirectional interactions, the interaction with higher predictability is indicated by a larger arrow; however, if the two interactions are similar in strength (i.e., overlapping predictability 95% CIs), then these are indicated by grey, equal‐sized arrows.
**Figure S9:** Human‐wildlife interactions detected by convergent cross mapping using the three‐camera‐trap dataset from 2010 to 2020. Shaded areas indicate 95% CIs of predictability (ρ), using the 2.5th and 97.5th percentiles from the 1000 bootstraps. Pink shading indicates if activity of humans influenced activity of animal, and green shading indicates if activity of animal influenced activity of humans. Blue and grey shading indicate results from the respective null models. No interactions were detected.
**Figure S10:** Optimal embedding dimension for the three‐camera‐trap dataset from 2010 to 2020. Simplex projection determined the optimal embedding dimension that best “unfolded” the dynamics, as measured by predictability (ρ). Optimal embedding dimension for each species, that was used for convergent cross mapping, is marked by a pink dashed line.
**Figure S11:** Evidence of nonlinear dynamics for the three‐camera‐trap dataset from 2010 to 2020. Sequential locally weighted global linear map (S‐map) forecasting method fitted local linear maps, with the localisation parameter (*θ*) assigning weights to points depending on their location in relation to the point to be predicted. When *θ* = 0, all points are weighted equally and the S‐map model corresponds to an autoregressive model (i.e., a linear model). When *θ* > 0, nearby points receive greater weights and the S‐map corresponds to a nonlinear model. For coyotes, deer and pumas, predictability (ρ) increased when *θ* > 0, thereby indicating nonlinear dynamics. At higher values of *θ*, predictability degrades as the S‐map model overfits.
**Figure S12:** Kernel density estimation of daily activity patterns under low and high puma activity. (a–f) Activity patterns of species outside (grey) and inside the preserve (green) during 2019–2022 (low and high puma activity across space). Overlap coefficient (∆) indicates similarity between activity patterns during low and high puma activity for each species (95% CIs in Table S4). *p* value indicates if the two activity patterns were different using the Watson's *U* ^2^ test (test statistics in Table S4). Where activity patterns are different, arrows indicate how activity patterns changed from low to high puma activity. *n* indicates the number of independent detections per year per camera for each sample. Activity patterns, under high puma activity, of species that pose predation and intraguild killing threats are displayed in dashed lines.
**Figure S13:** Kernel density estimation of human daily activity patterns under low and high puma activity. (a) Activity patterns of humans during November 2010–December 2012 (grey) and January 2015–March 2020 (green) at Jasper Ridge Biological Preserve ('Ootchamin 'Ooyakma). (b) Activity patterns of humans outside (grey) and inside the preserve (green) during July 2019–December 2022. Overlap coefficient (∆) indicates similarity between activity patterns during low and high puma activity (95% CIs in Tables S3 and S4). *p* value indicates if the two activity patterns were different using the Watson's *U* ^2^ test (test statistics in Tables S3 and S4). Where activity patterns are different, arrows indicate how activity patterns changed from low to high puma activity. While the Watson's *U* ^2^ statistics indicated changes, the range of hours for human activity remained similar (predominantly diurnal). *n* indicates number of independent detections per year per camera for each sample.
**Figure S14:** Vegetation composition in quadrats sampled in both 2006 and 2023. (a) Plant cover estimated from 10 × 10 m quadrats, using the line intercept method. Statistically significant difference between years was evaluated using a Chi‐squared test. (b) Woody plant density estimated by counting woody plants with basal diameter ≥ 1 cm in each of the sampled 10 × 10 m quadrats. Statistically significant difference between years was evaluated using paired Wilcoxon signed‐rank test. In a–b, error bars indicate 95% CIs and Holm–Bonferroni corrections are applied to *p* values. (c) Distribution of basal diameters of woody plants with basal diameters ≥ 1 cm and ≤ 20 cm. In a–c, grey indicates year with low puma activity (2006) and green indicates year with high puma activity (2023).
**Figure S15:** Change in vegetation over time. Each photograph captures the same location in May of 2012 (a), 2016 (b) and 2023 (c). Density of woody plants increases over time, particularly in the background.
**Figure S16:** Deer herbivory increases mortality of coast live oak ( *Quercus agrifolia* ) saplings. In 2009 (Year 0), 75 oak saplings were individually caged at the preserve to exclude deer herbivory and paired with 75 adjacent uncaged saplings (mean pairwise distance = 1.4 m) exposed to herbivory. The matched‐pair design allowed for direct comparison of survival over time. Shaded bands represent 95% confidence intervals. Data were provided from a separate, ongoing study at the preserve. Over five years, deer herbivory reduced sapling survival by 26%, providing causal evidence that deer can suppress woody plant recruitment.
**Figure S17:** Cumulative precipitation over past two years of each vegetation survey. Data are sourced from the National Oceanic and Atmospheric Administration and are based on weather stations in Woodside, California.
**Figure S18:** Average daily temperature over past one year of each vegetation survey. Data (thin lines) are sourced from the National Oceanic and Atmospheric Administration and are based on weather stations in La Honda, California. Thick lines are loess curves, with grey shading indicating 95% CIs.
**Figure S19:** Black‐tailed deer carcasses recorded on California Roadkill Observation System. Each point indicates an observation of roadkill recorded by citizen scientists or the California Highway Patrol (Waetjen and Shilling 2017). Green and blue lines outline the boundaries of the preserve and adjacent monitored area respectively, with solid lines indicating fenced boundaries. White dashed lines indicate trails. Yellow lines indicate the Interstate 280 highway.
**Table S1:** Convergent cross mapping results using the seventeen‐camera‐trap dataset from 2012 to 2020. Mean predictability (ρ) was calculated from 1000 bootstraps using the full time series (length of time series = 2326 days), and 95% CIs of predictabilities were from the 2.5th and 97.5th percentiles of bootstraps.
**Table S2:** Convergent cross mapping results using the three‐camera‐trap dataset from 2010 to 2020. Mean predictability (ρ) was calculated from 1000 bootstraps using the full time series (length of time series = 3057 days), and 95% CIs of predictabilities were from the 2.5th and 97.5th percentiles of bootstraps.
**Table S3:** Summary statistics from comparing daily activity patterns of wildlife across low and high puma activity across time (Figure 3a–f). n indicates number of independent detections per year per camera for under low and high puma activity. Overlap indicates overlap coefficient of activity patterns during low and high puma activity for each species, with 95% CIs calculated from 1000 bootstraps. Watson's *U* ^2^ test and associated *p* value indicate if the activity patterns of the species were different between low and high puma activity.
**Table S4:** Summary statistics from comparing daily activity patterns of wildlife across low and high puma activity across space (Figure S12a–f). *n* indicates number of independent detections per year per camera for under low and high puma activity. Overlap indicates overlap coefficient of activity patterns during low and high puma activity for each species, with 95% CIs calculated from 1000 bootstraps. Watson's *U* ^2^ test and associated *p* value indicate if the activity patterns of the species were different between low and high puma activity.
**Table S5:** Changes in activity pattern overlap between species across low and high puma activity across time (Figure 3a–f). Overlap (without change) indicates the overlap coefficient (with 95% CIs calculated from 1000 bootstraps) between Species A under low puma activity and Species B under high puma activity. Overlap (with change) indicates the overlap coefficient (with 95% CIs calculated from 1000 bootstraps) between Species A and B under high puma activity. Only species which significantly shifted their daily activity patterns are assessed here.
**Table S6:** Changes in activity pattern overlap between species across low and high puma activity across space (Figure S12a–f). Overlap (without change) indicates the overlap coefficient (with 95% CIs calculated from 1000 bootstraps) between Species A under low puma activity and Species B under high puma activity. Overlap (with change) indicates the overlap coefficient (with 95% CIs calculated from 1000 bootstraps) between Species A and B under high puma activity. Only species which significantly shifted their daily activity patterns are assessed here.
**Table S7:** Precipitation and temperature data for each year that vegetation was sampled. Data are sourced from the National Oceanic and Atmospheric Administration.

## Data Availability

Camera‐trap data, plant sampling data and simplified R code to reproduce CCM results are available at Sonawane et al. (2026).
