# Supplementary material for: Mammal Community Responses to Increasing Puma Activity in a Suburban Preserve
Source: Ecol Evol. 2026 Jun 17;16(6):e73775. doi: 10.1002/ece3.73775 (PMC13274232; doi:10.1002/ece3.73775)
Supplement: Supplementary file 1 — Figure S1: Study area. (a) Location of Jasper Ridge Biological Preserve ('Ootchamin 'Ooyakma) in the Bay Area, California, USA. Purple polygon indicates the area of the preserve. The preserve sits in between the suburbia of the Bay Area and the Santa Cruz Mountains. Areas highlighted in pink indicate presence of human populations (Meta 2020). (b) Locations of the cameras and vegetation quadrats inside and outside the preserve. Colours in image have been enhanced to better delineate oak woodland and grassland habitats. Note building structures around preserve, indicating presence of people. Figure S2: Change in activity of mammals at Jasper Ridge Biological Preserve ('Ootchamin 'Ooyakma) from the camera‐trap datasets. Time series of activity (standardised moving sum of independent detections per camera over a 365‐day window) from three cameras in November 2010 to March 2020 (“long time series”, in green) and from 17 cameras in November 2012 to March 2020 (“short time series”, in black). Respective n values indicate the total number of independent detections for each species for each of the two datasets. Figure S3: Puma kittens at Jasper Ridge Biological Preserve ('Ootchamin 'Ooyakma) in 2013 (a), 2015 (b), 2016 (c, d) and 2018 (e). Figure S4: Wildlife interactions detected by convergent cross mapping using the seventeen‐camera‐trap dataset from 2012 to 2020. Shaded areas indicate 95% CIs of predictability (ρ), using the 2.5th and 97.5th percentiles from the 1000 bootstraps. Pink shading indicates if activity of species on the left influenced that on the right, and green shading indicates if activity of species on the right influenced that on the left. Blue and grey shading indicate results from the respective null models. Pink arrow indicates left species significantly influenced right species (predictability of empirical model > predictability of null model), while green arrow indicates right species significantly influenced left species. In bidirectional interactio [file ECE3-16-e73775-s001.docx]

**
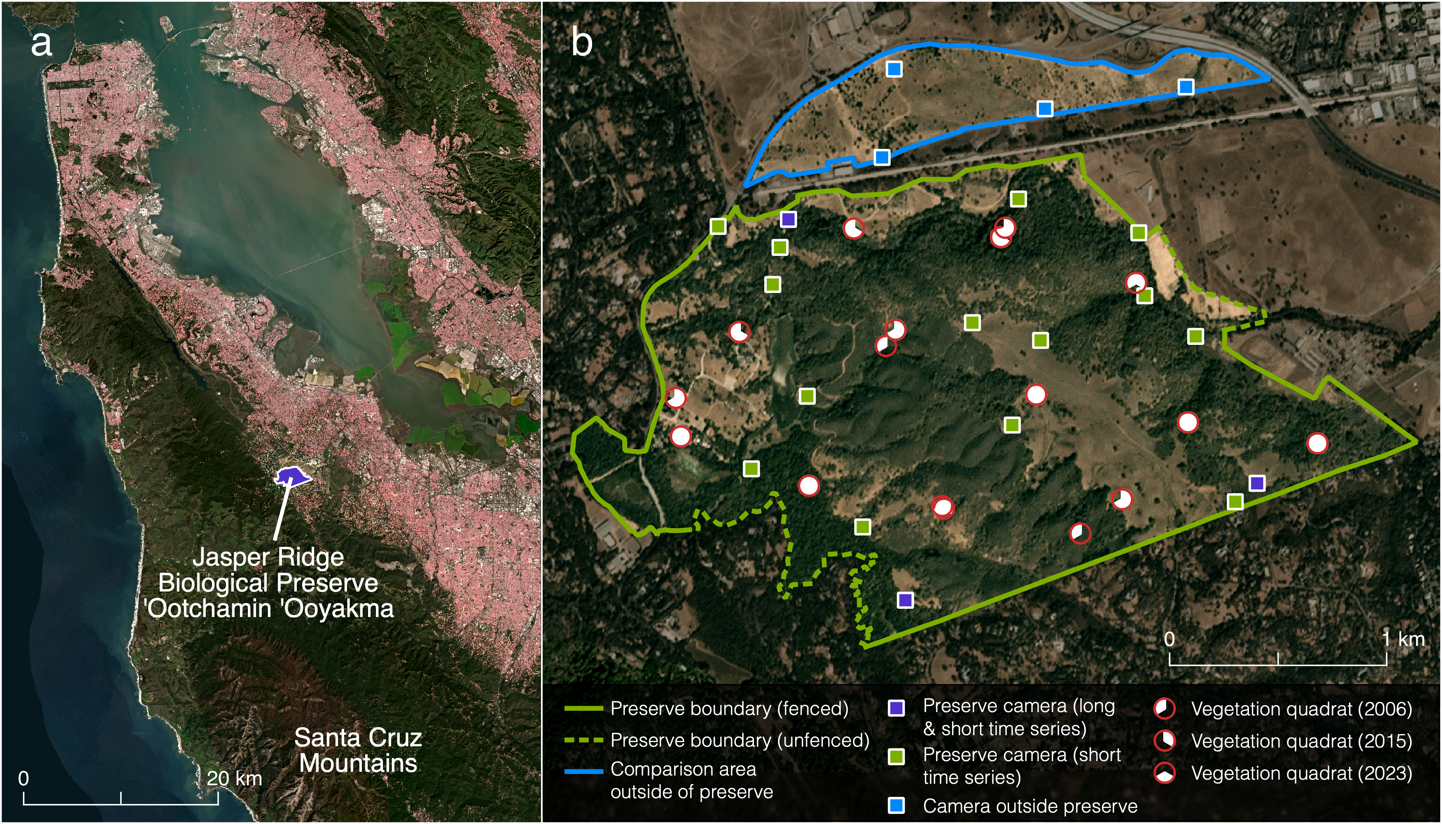
**

**Fig. S1: Study area. a**, Location of Jasper Ridge Biological Preserve ('Ootchamin 'Ooyakma) in the Bay Area, California, USA. Purple polygon indicates the area of the preserve. The preserve sits in between the suburbia of the Bay Area and the Santa Cruz Mountains. Areas highlighted in pink indicate presence of human populations (Meta, 2020). **b**, Locations of the cameras and vegetation quadrats inside and outside the preserve. Colours in image have been enhanced to better delineate oak woodland and grassland habitats. Note building structures around preserve, indicating presence of people.

**
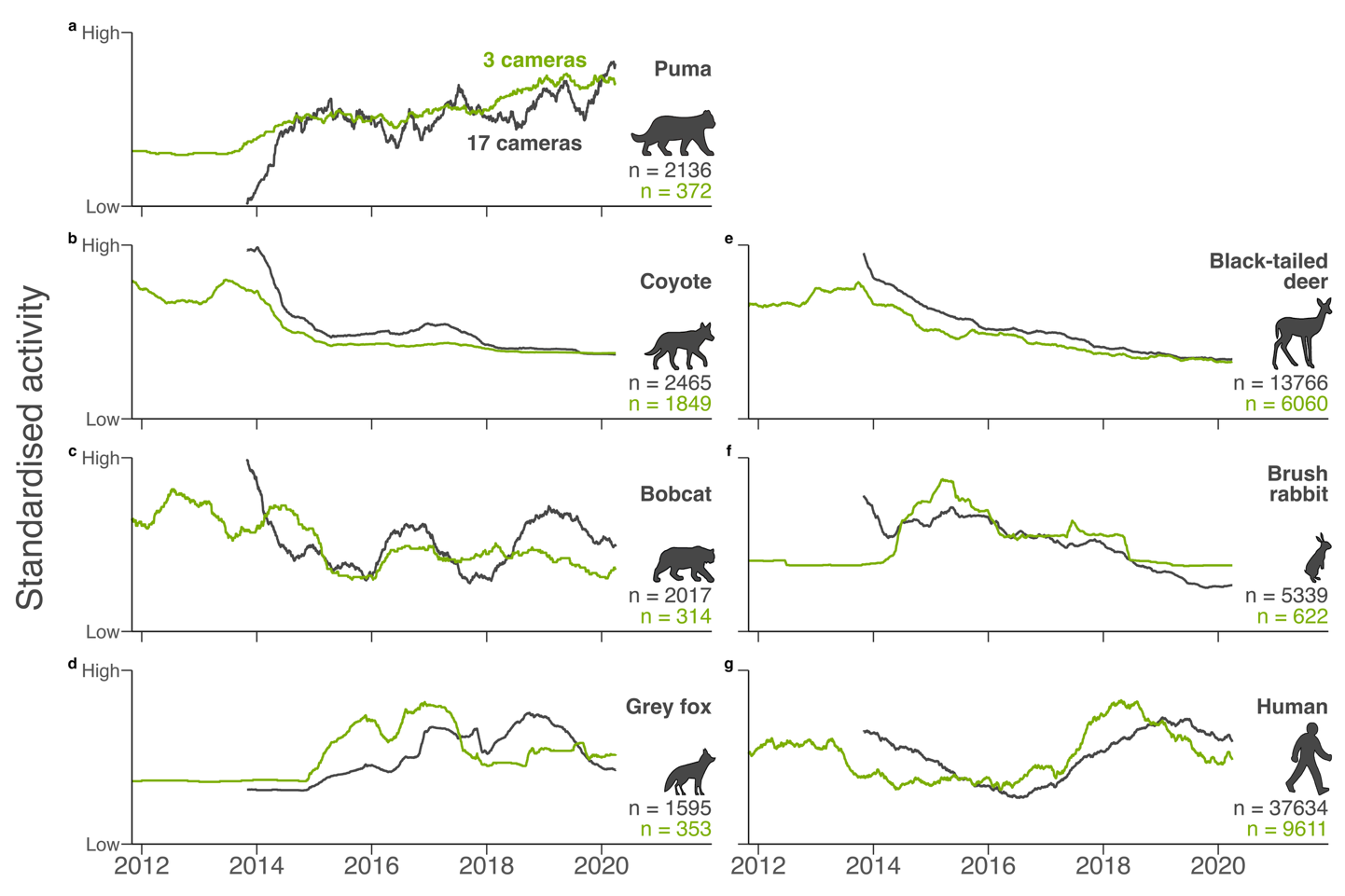
**

**Fig. S2: Change in activity of mammals at Jasper Ridge Biological Preserve ('Ootchamin 'Ooyakma) from the camera-trap datasets.** Time series of activity (standardised moving sum of independent detections per camera over a 365-day window) from three cameras in November 2010 to March 2020 (“long time series”, in green) and from 17 cameras in November 2012 to March 2020 (“short time series”, in black). Respective n values indicate the total number of independent detections for each species for each of the two datasets.

**
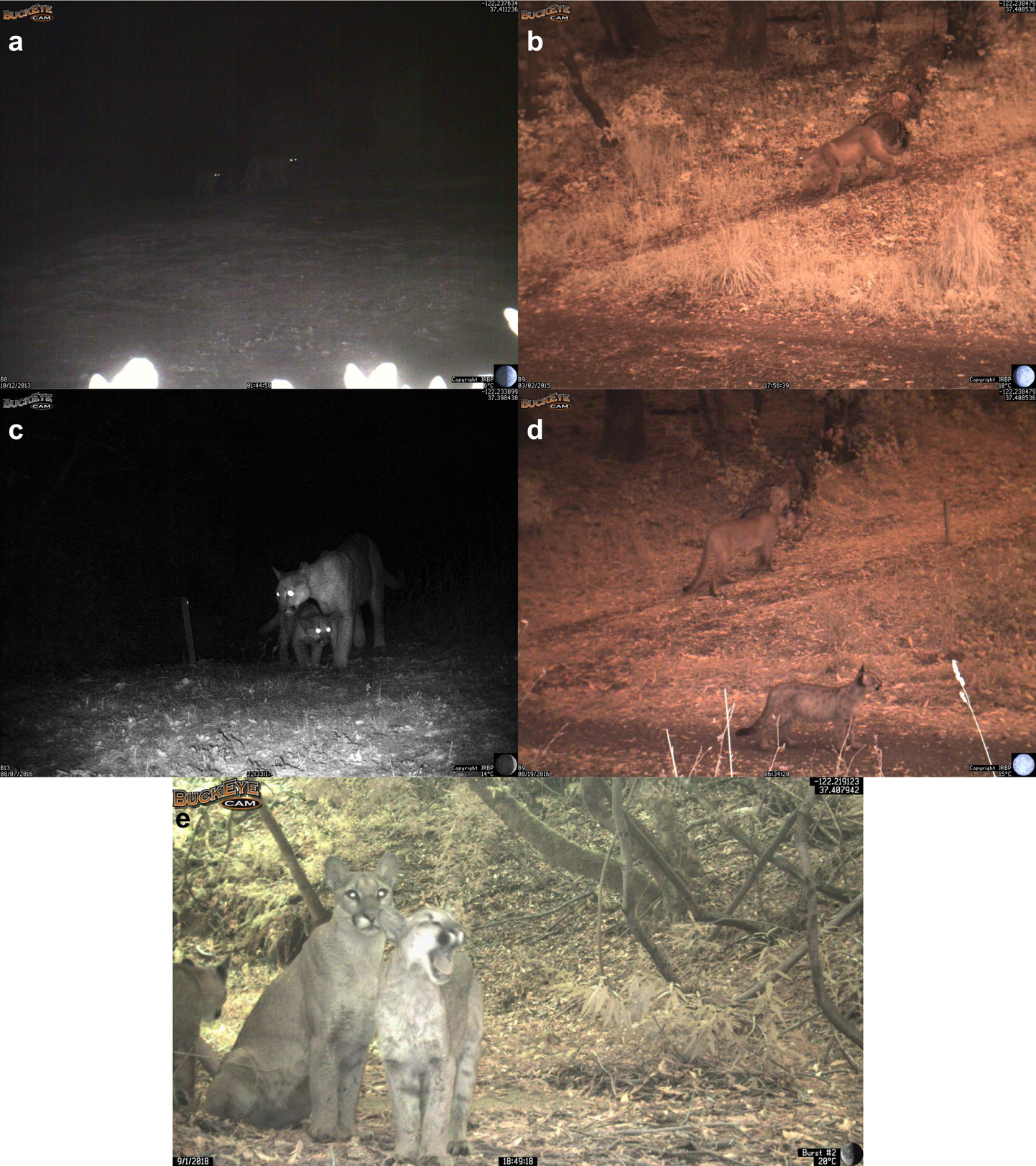
**

**Fig. S3: Puma kittens at Jasper Ridge Biological Preserve ('Ootchamin 'Ooyakma) in 2013 (a), 2015 (b), 2016 (c-d) and 2018 (e).**

**Table S1: Convergent cross mapping results using the seventeen-camera-trap dataset from 2012 to 2020.** Mean predictability ($\rho$) was calculated from 1000 bootstraps using the full time series (length of time series = 2326 days), and 95% CIs of predictabilities were from the 2.5^th^ and 97.5^th^ percentiles of bootstraps.
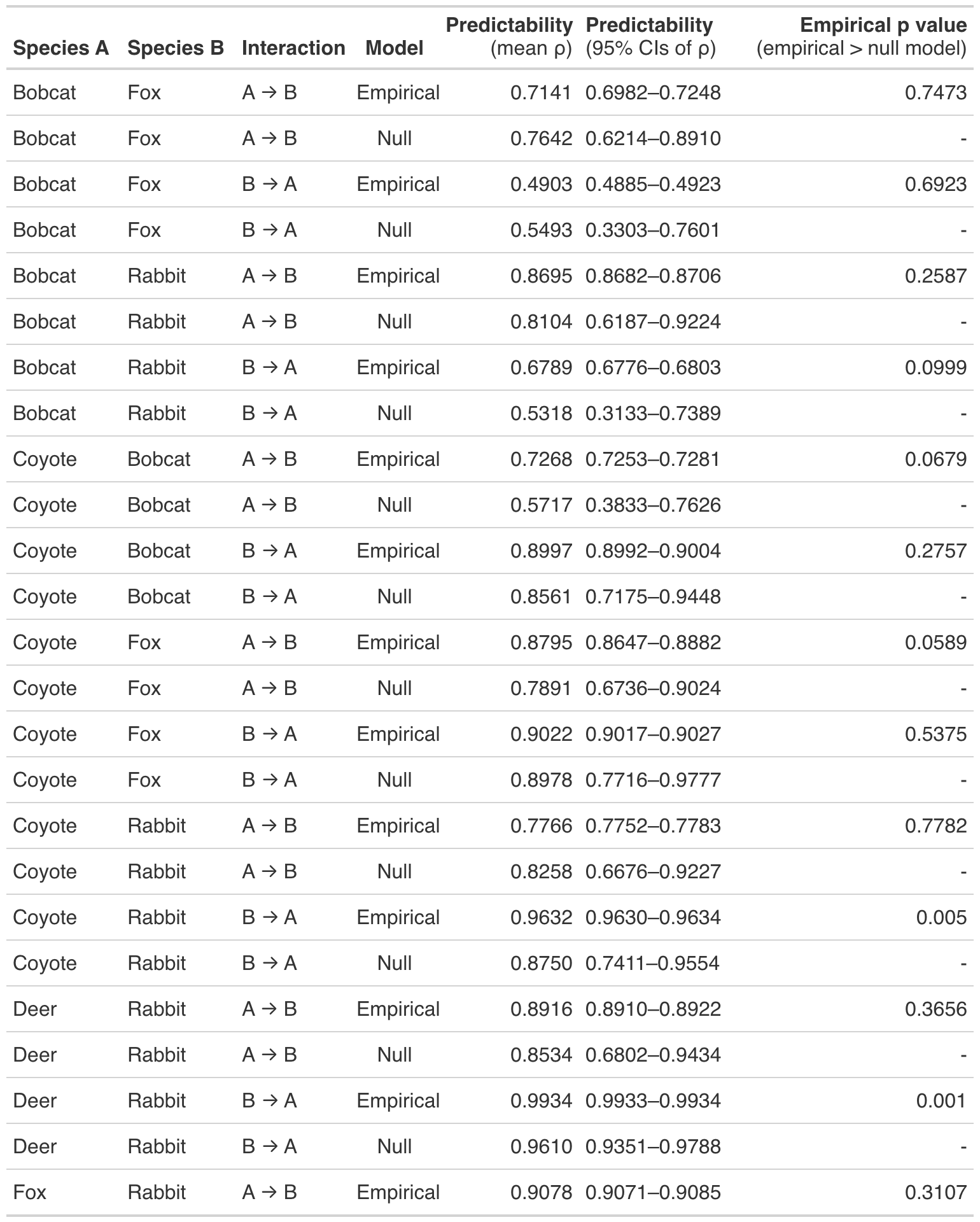

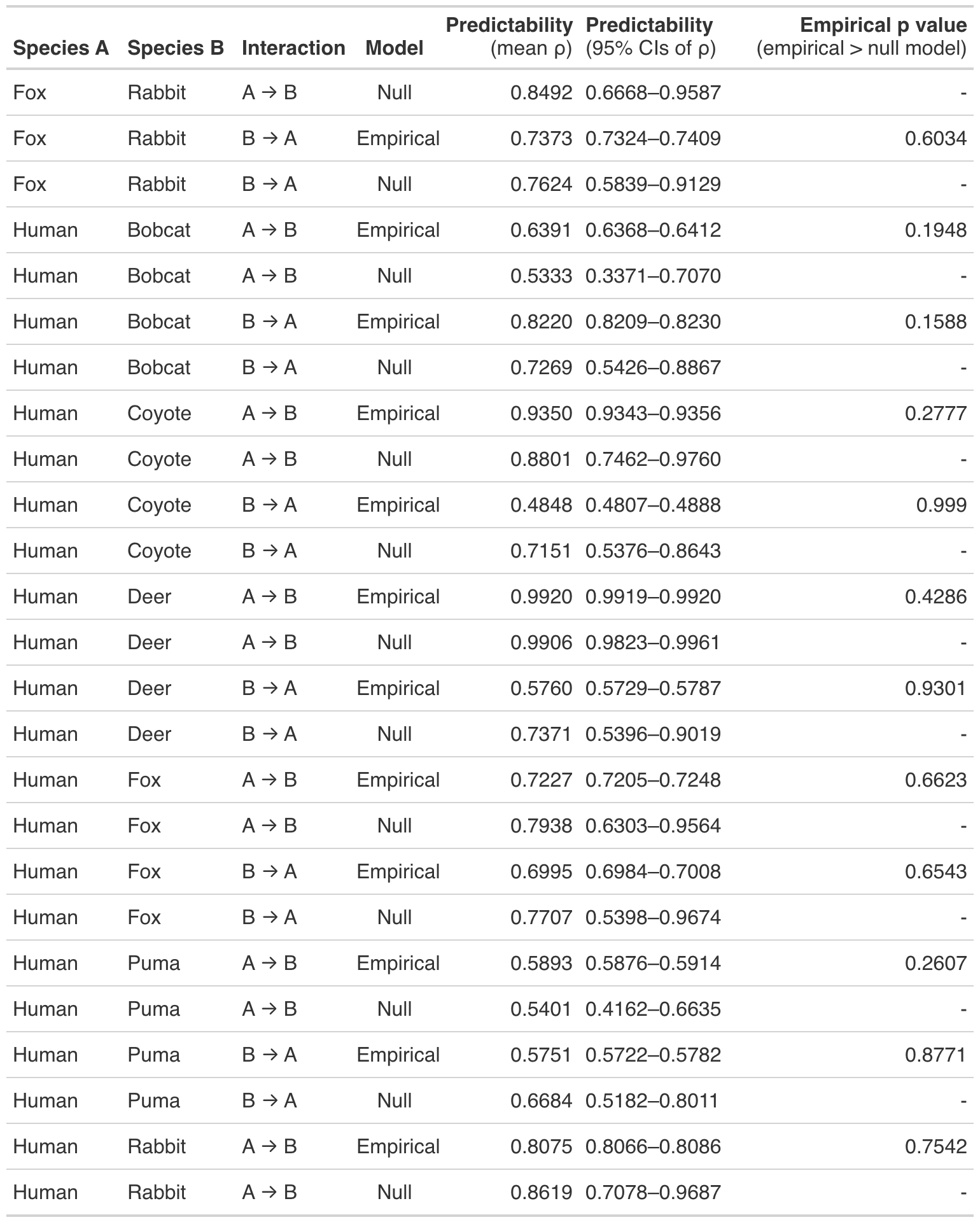

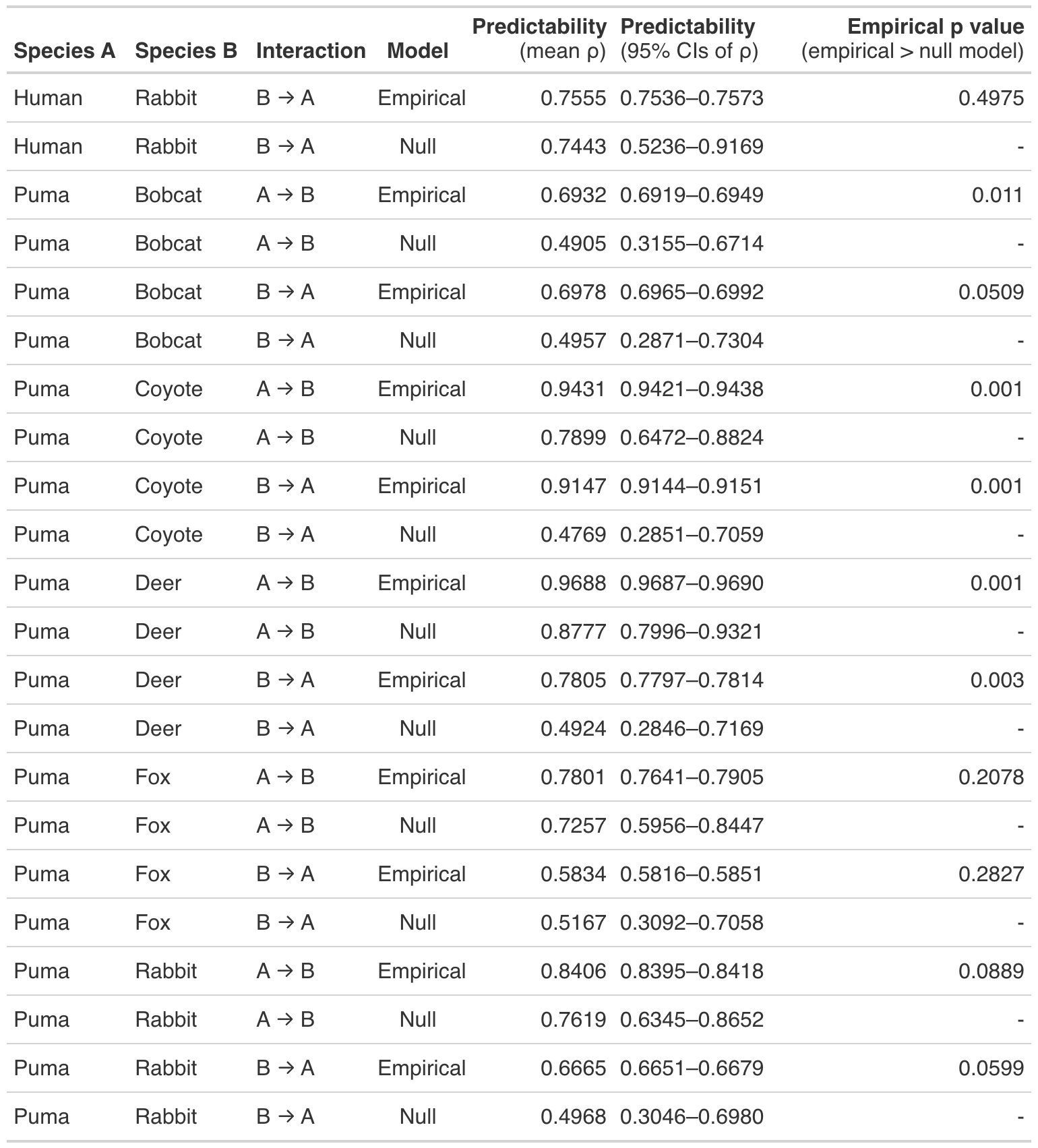


**
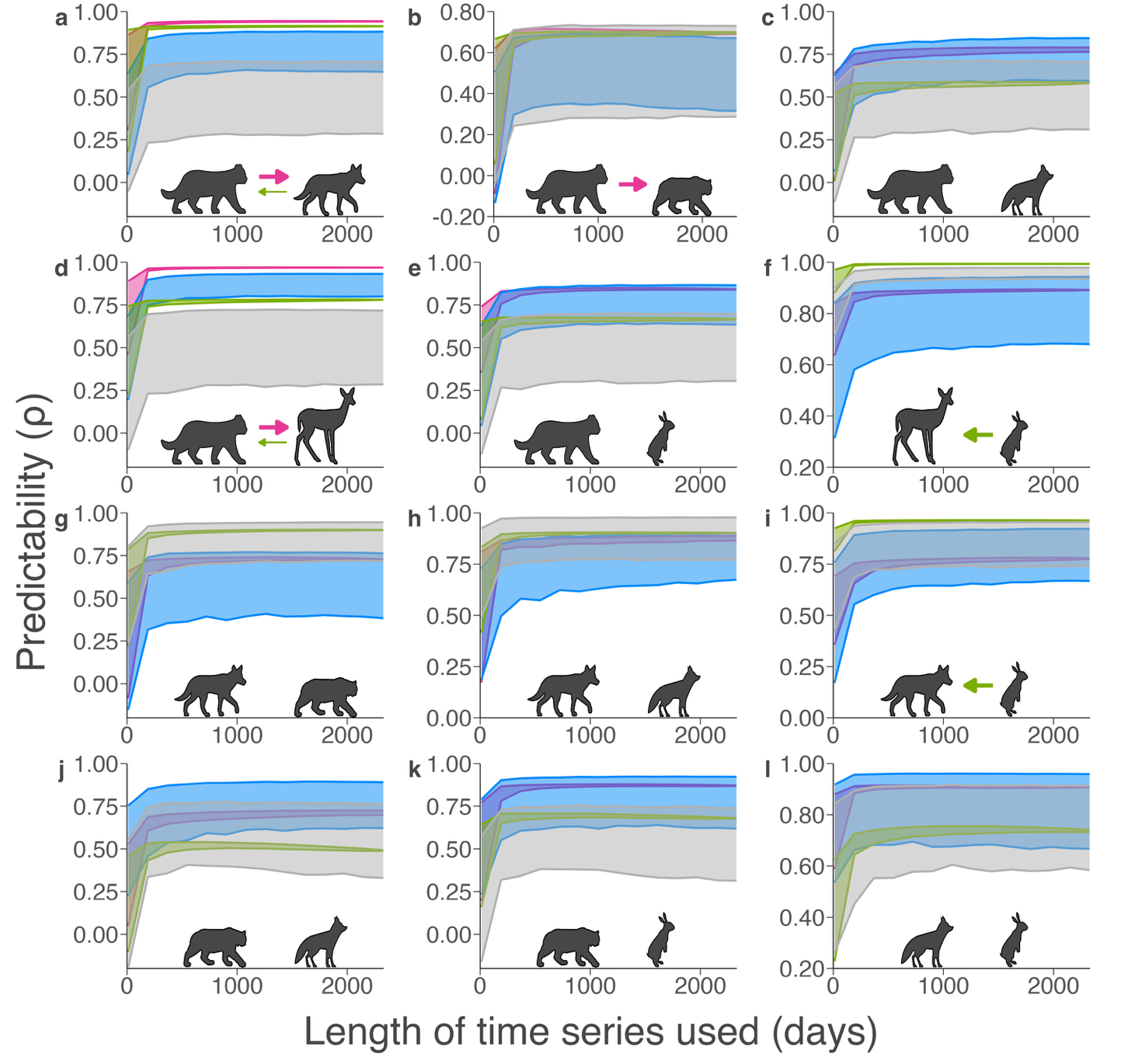
**

**Fig. S4: Wildlife interactions detected by convergent cross mapping using the seventeen-camera-trap dataset from 2012 to 2020.** Shaded areas indicate 95% CIs of predictability ($\rho$), using the 2.5^th^ and 97.5^th^ percentiles from the 1000 bootstraps. Pink shading indicates if activity of species on the left influenced that on the right, and green shading indicates if activity of species on the right influenced that on the left. Blue and grey shading indicate results from the respective null models. Pink arrow indicates left species significantly influenced right species (predictability of empirical model > predictability of null model), while green arrow indicates right species significantly influenced left species. In bidirectional interactions, the interaction with higher predictability is indicated by a larger arrow.


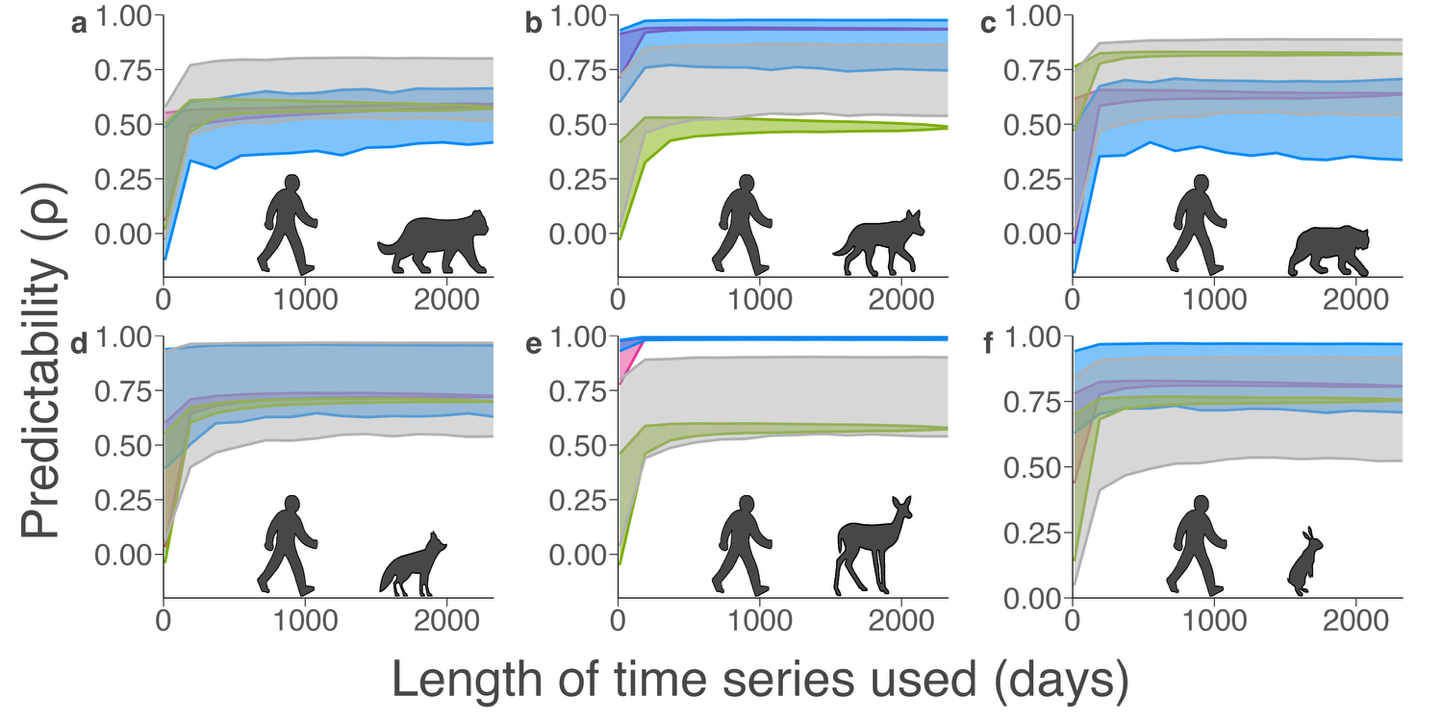


**Fig. S5: Human-wildlife interactions detected by convergent cross mapping using the seventeen-camera-trap dataset from 2012 to 2020.** Shaded areas indicate 95% CIs of predictability ($\rho$), using the 2.5^th^ and 97.5^th^ percentiles from the 1000 bootstraps. Pink shading indicates if activity of humans influenced activity of animal, and green shading indicates if activity of animal influenced activity of humans. Blue and grey shading indicate results from the respective null models. No interactions were detected.

**
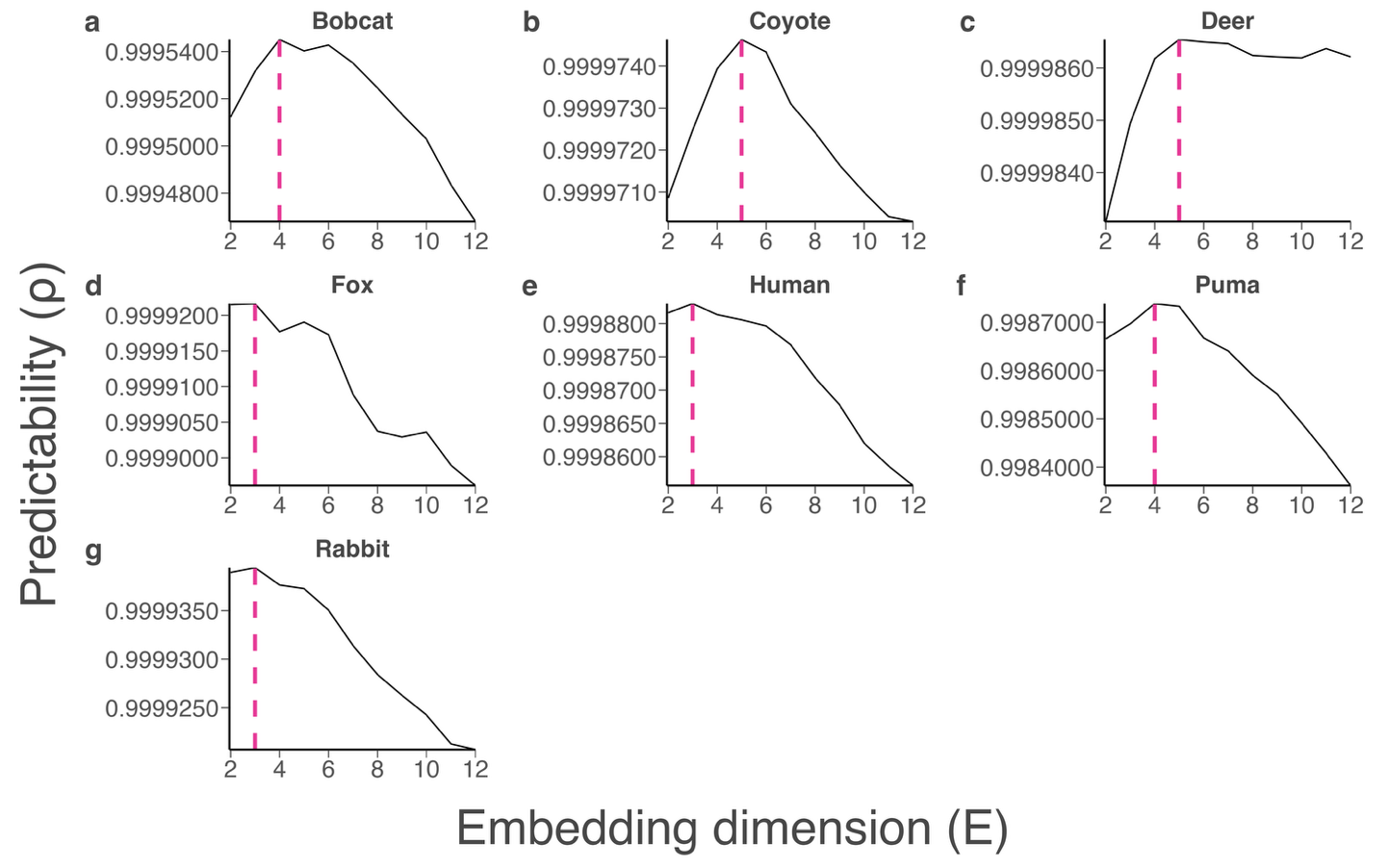
**

**Fig. S6: Optimal embedding dimension for the seventeen-camera-trap dataset from 2012 to 2020.** Simplex projection determined the optimal embedding dimension that best “unfolded” the dynamics, as measured by predictability ($\rho$). Optimal embedding dimension for each species, that was used for convergent cross mapping, is marked by a pink dashed line.


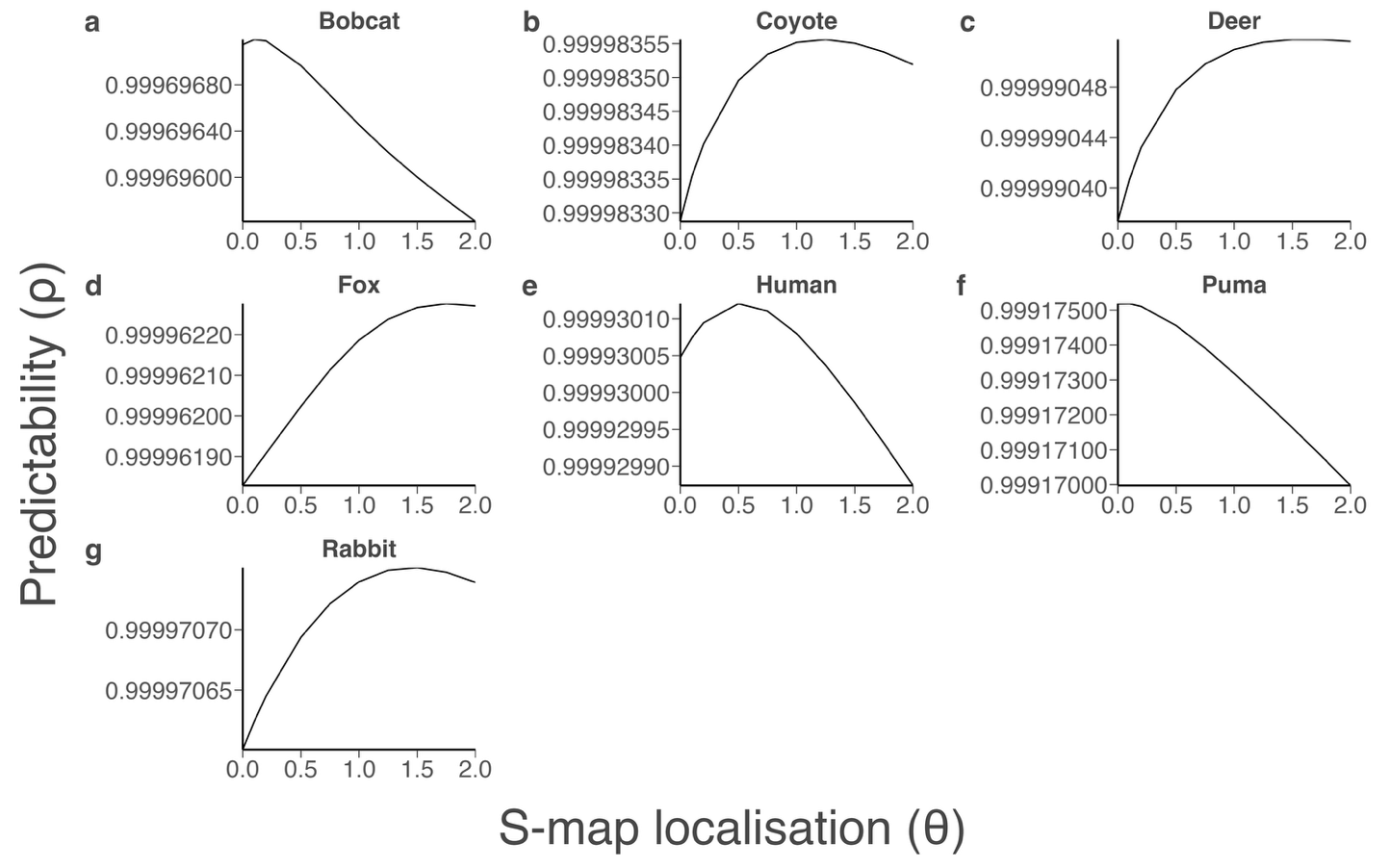


**Fig. S7: Evidence of nonlinear dynamics for the seventeen-camera-trap dataset from 2012 to 2020.** Sequential locally weighted global linear map (S-map) forecasting method fitted local linear maps, with the localisation parameter (θ) assigning weights to points depending on their location in relation to the point to be predicted. When θ = 0, all points are weighted equally, and the S-map model corresponds to an autoregressive model (i.e., a linear model). When θ > 0, nearby points receive greater weights, and the S-map corresponds to a nonlinear model. For bobcats, coyotes, deer, foxes, humans, and rabbits, predictability ($\rho$) increased when θ > 0, thereby indicating nonlinear dynamics. At higher values of θ, predictability degrades as the S-map model overfits.

**Table S2: Convergent cross mapping results using the three-camera-trap dataset from 2010 to 2020.** Mean predictability ($\rho$) was calculated from 1000 bootstraps using the full time series (length of time series = 3057 days), and 95% CIs of predictabilities were from the 2.5^th^ and 97.5^th^ percentiles of bootstraps.
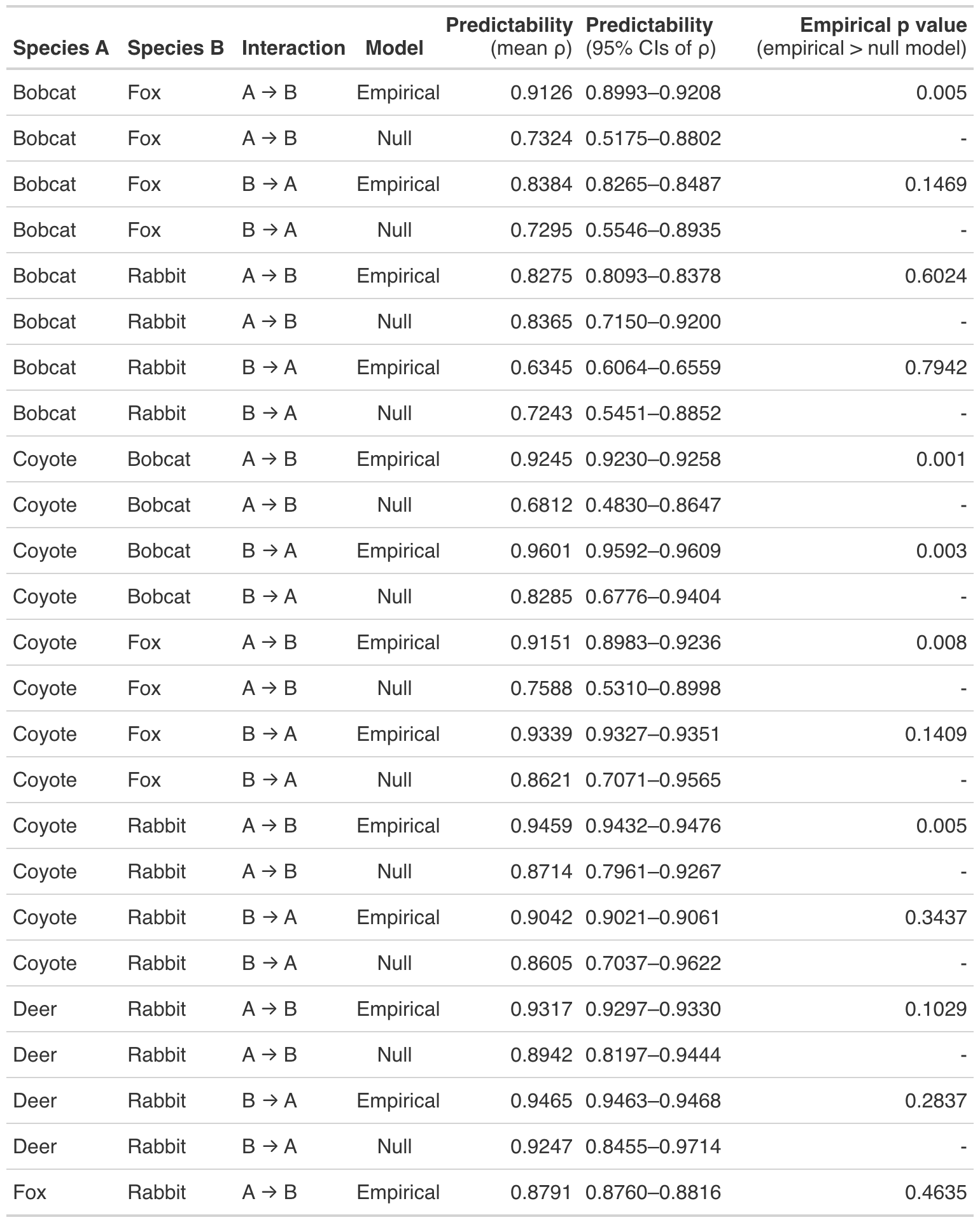

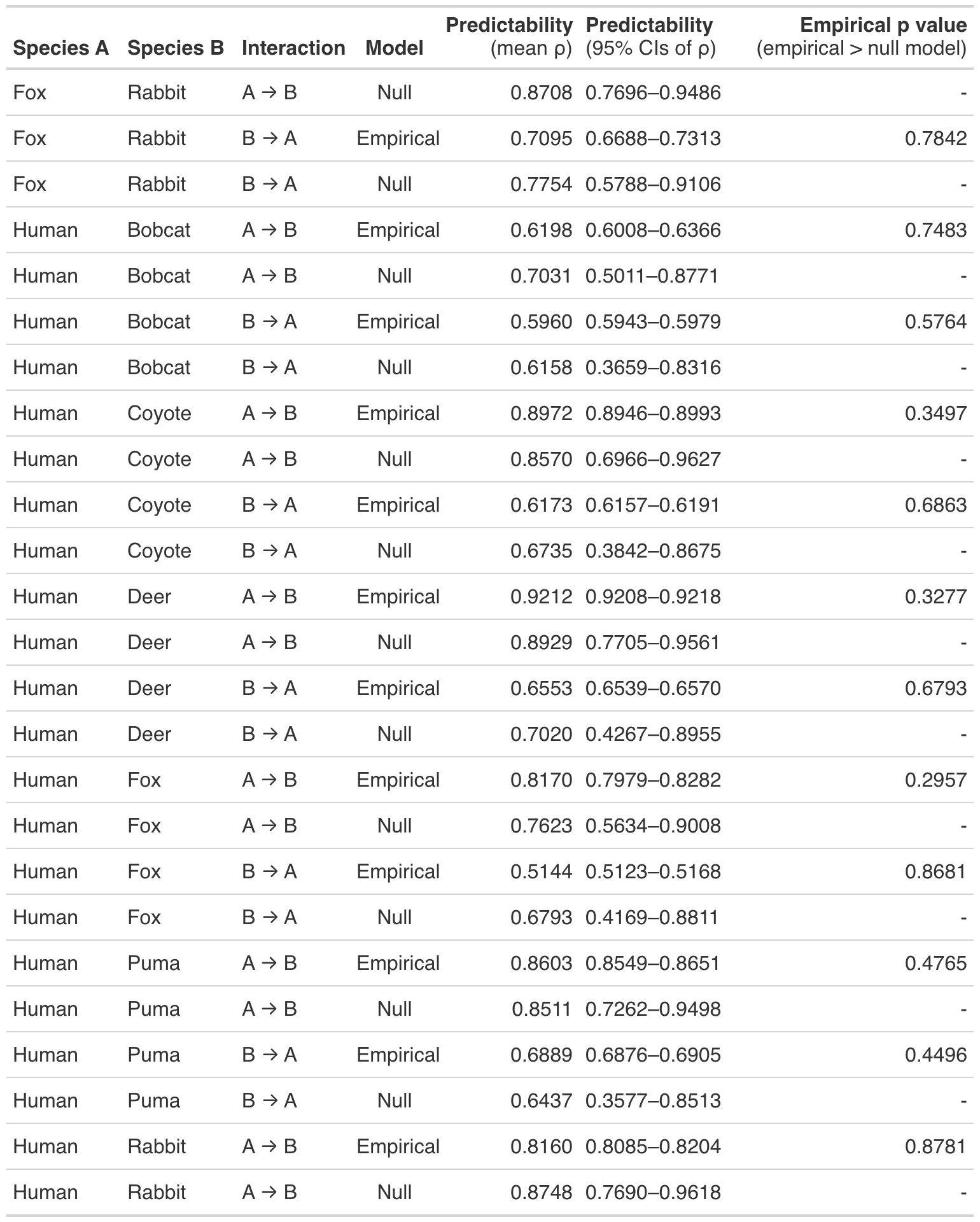

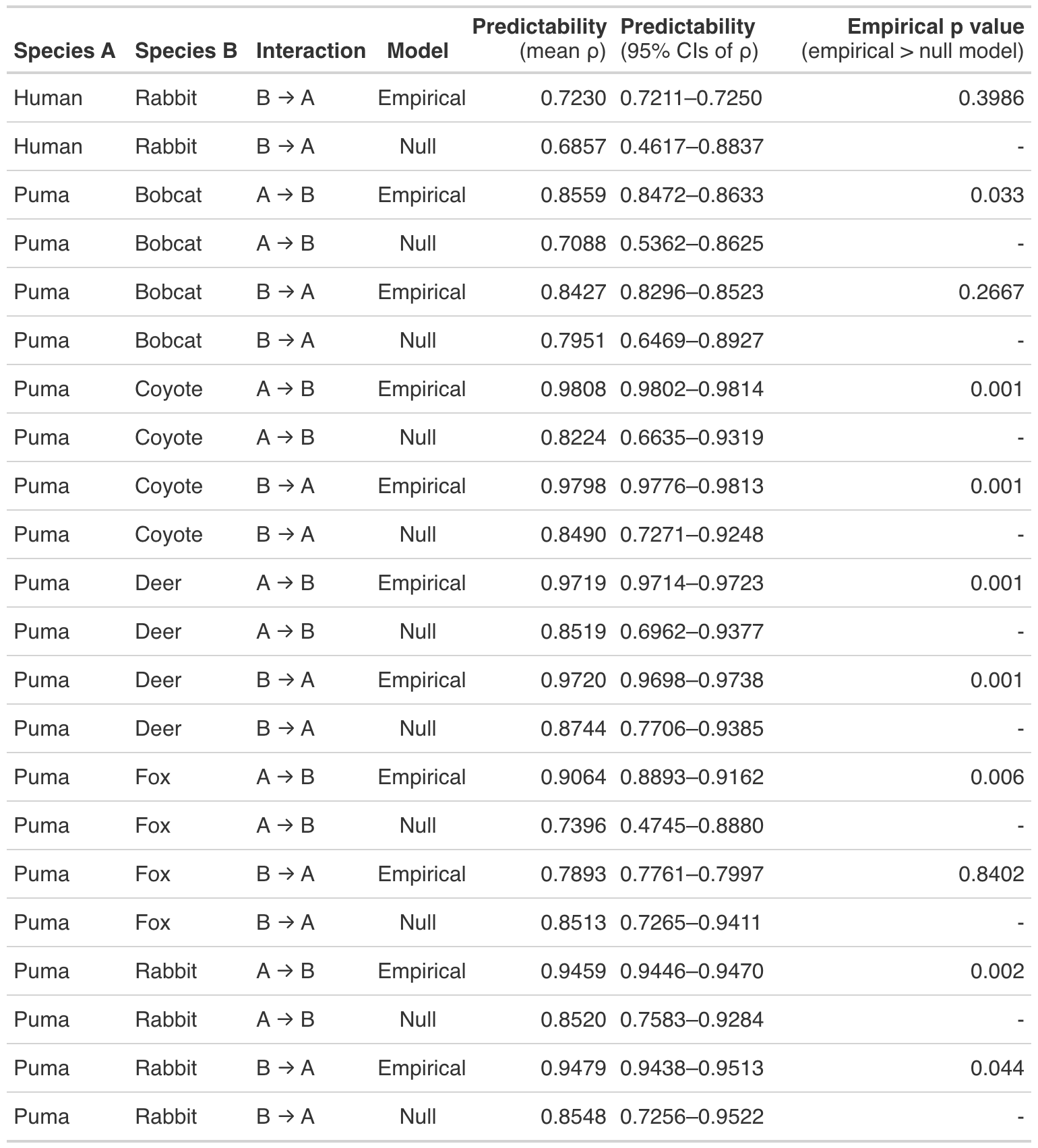


**
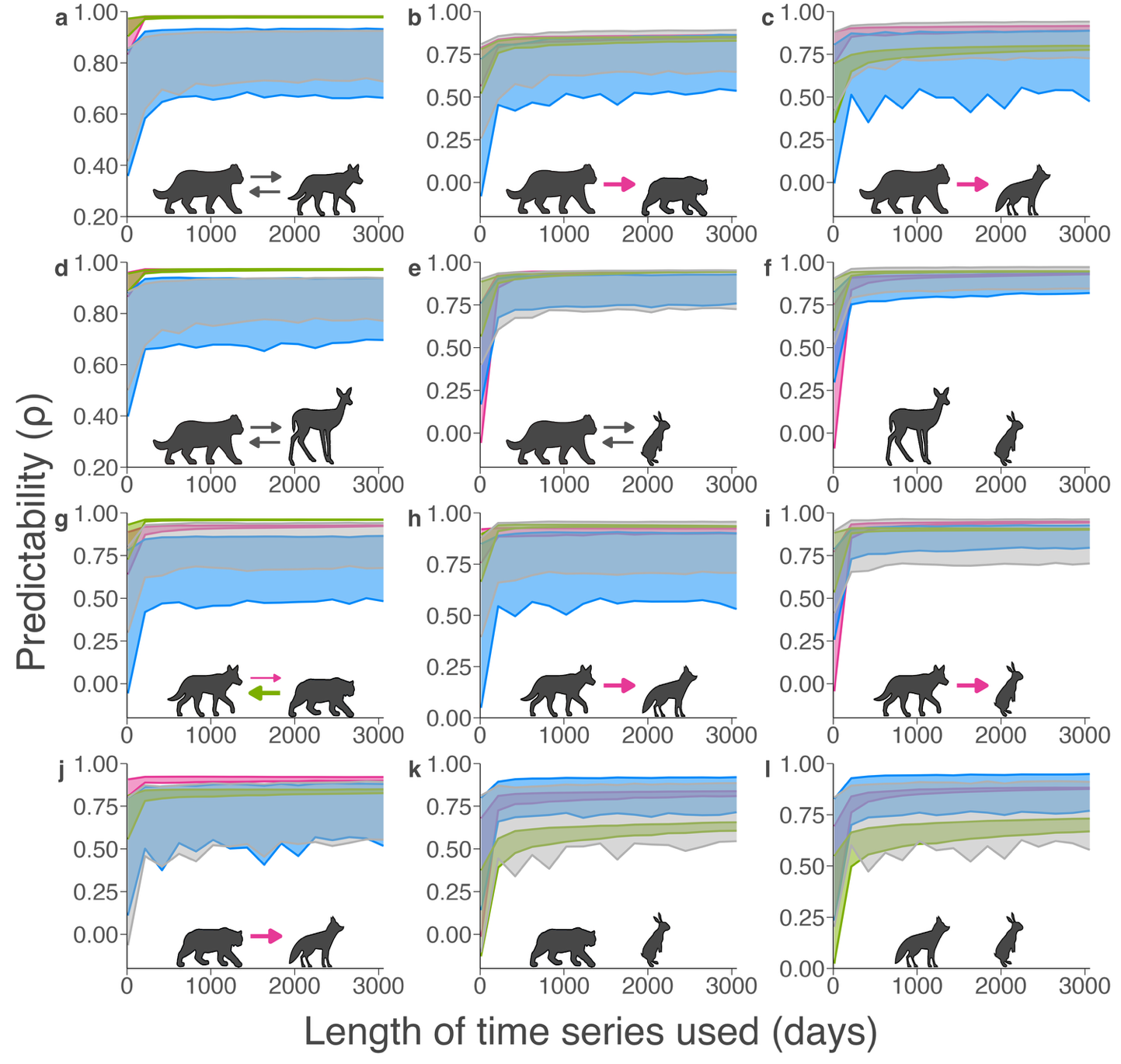
**

**Fig. S8: Wildlife interactions detected by convergent cross mapping using the three-camera-trap dataset from 2010 to 2020.** Shaded areas indicate 95% CIs of predictability ($\rho$), using the 2.5^th^ and 97.5^th^ percentiles from the 1000 bootstraps. Pink shading indicates if activity of species on the left influenced that on the right, and green shading indicates if activity of species on the right influenced that on the left. Blue and grey shading indicate results from the respective null models. Pink arrow indicates left species significantly influenced right species (predictability of empirical model > predictability of null model), while green arrow indicates right species significantly influenced left species. In bidirectional interactions, the interaction with higher predictability is indicated by a larger arrow; however, if the two interactions are similar in strength (i.e., overlapping predictability 95% CIs), then these are indicated by grey, equal-sized arrows.


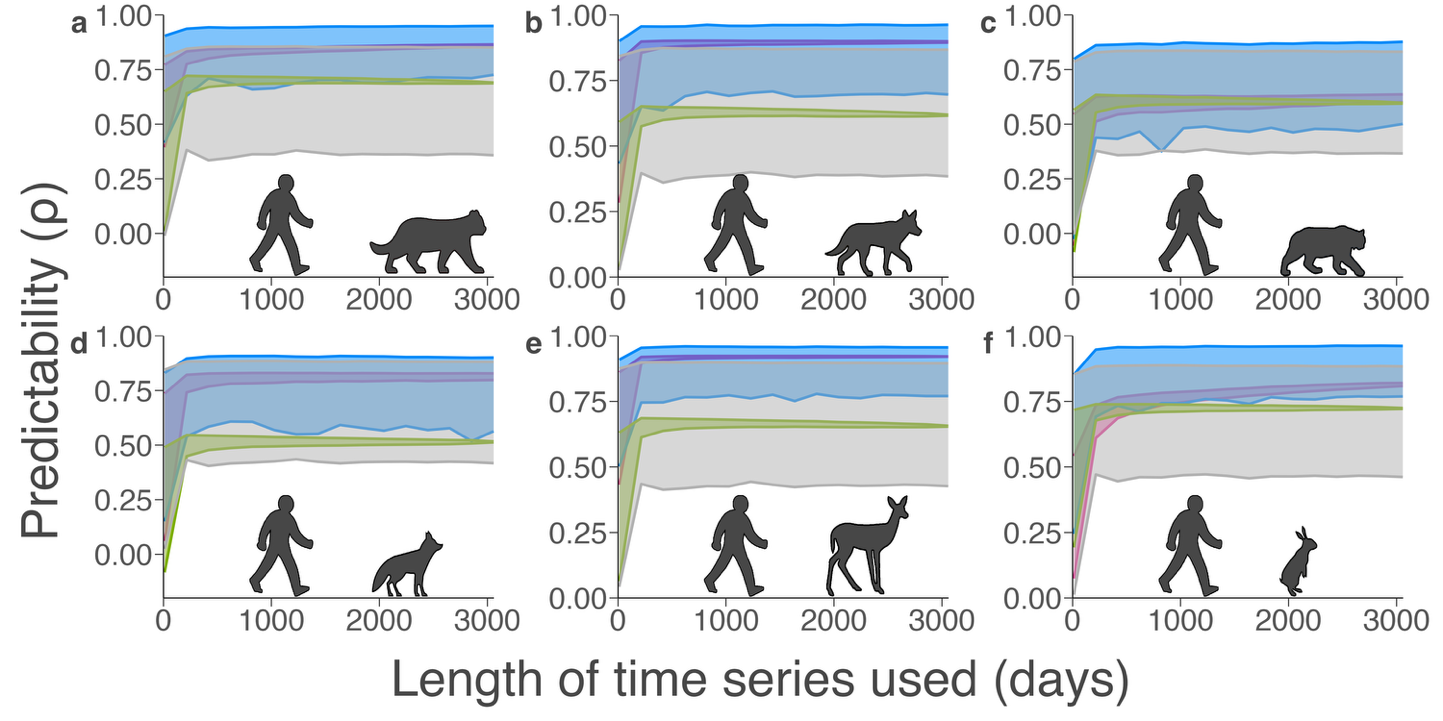


**Fig. S9: Human-wildlife interactions detected by convergent cross mapping using the three-camera-trap dataset from 2010 to 2020.** Shaded areas indicate 95% CIs of predictability ($\rho$), using the 2.5^th^ and 97.5^th^ percentiles from the 1000 bootstraps. Pink shading indicates if activity of humans influenced activity of animal, and green shading indicates if activity of animal influenced activity of humans. Blue and grey shading indicate results from the respective null models. No interactions were detected.

**
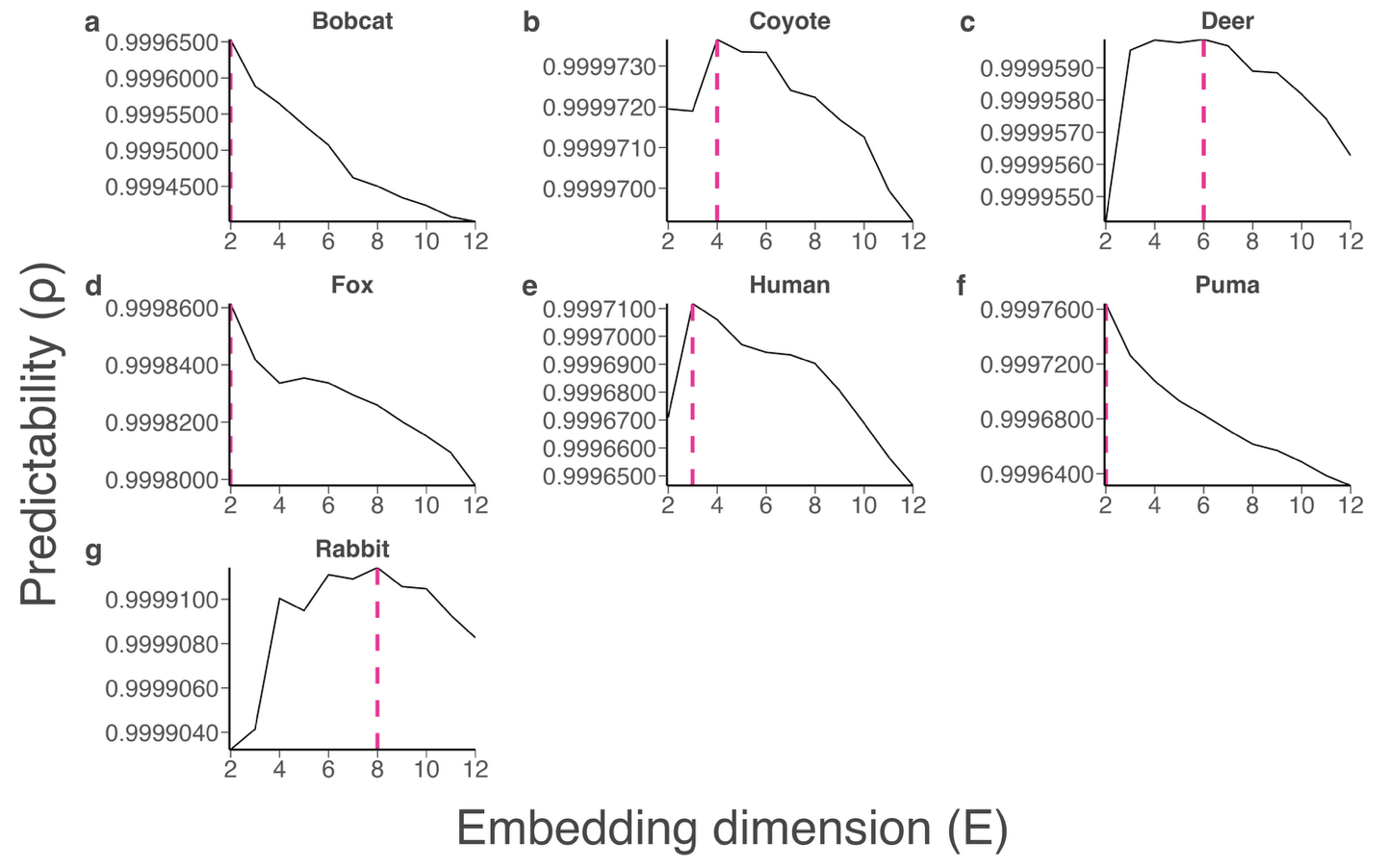
**

**Fig. S10: Optimal embedding dimension for the three-camera-trap dataset from 2010 to 2020.** Simplex projection determined the optimal embedding dimension that best “unfolded” the dynamics, as measured by predictability ($\rho$). Optimal embedding dimension for each species, that was used for convergent cross mapping, is marked by a pink dashed line.


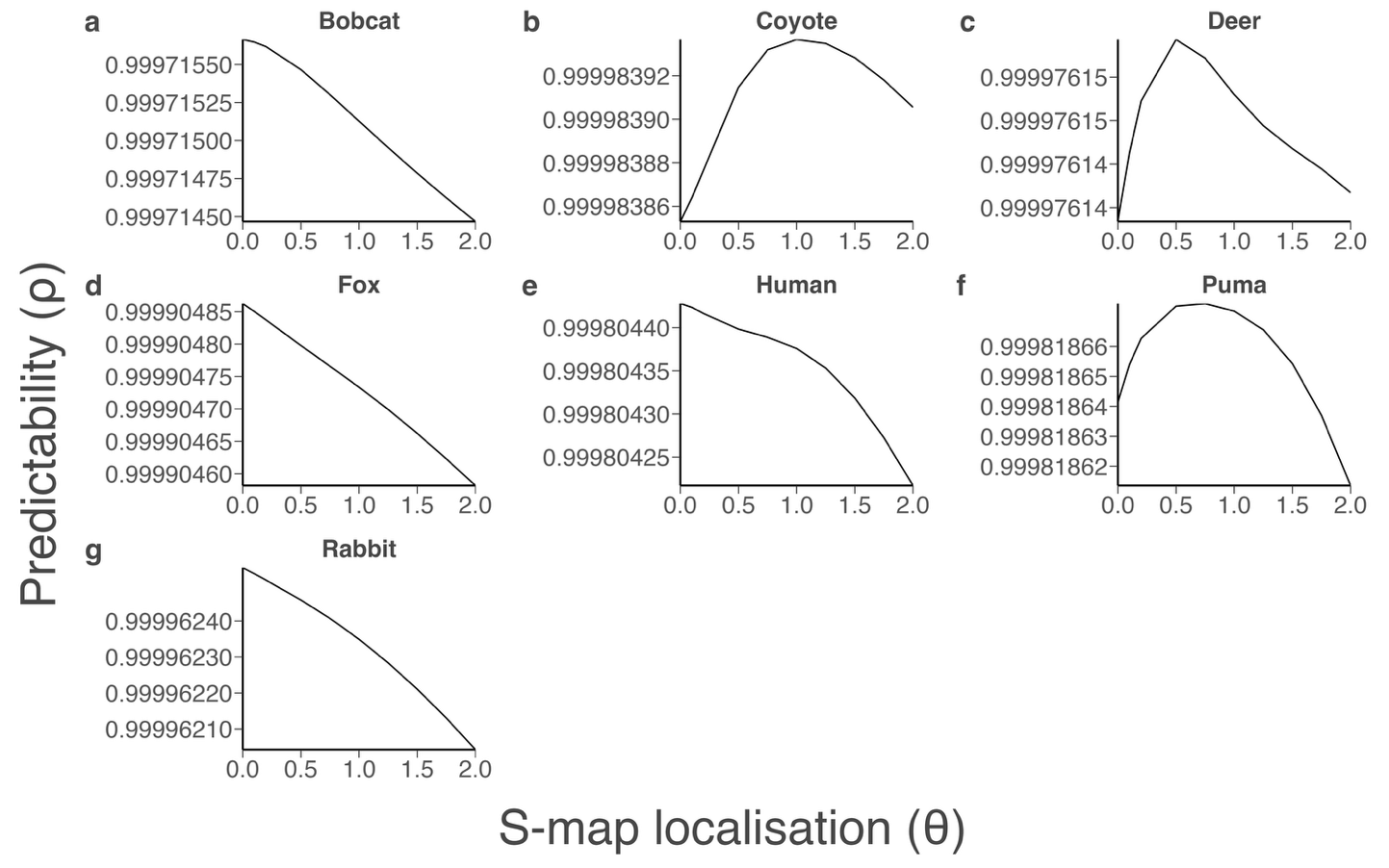


**Fig. S11: Evidence of nonlinear dynamics for the three-camera-trap dataset from 2010 to 2020.** Sequential locally weighted global linear map (S-map) forecasting method fitted local linear maps, with the localisation parameter (θ) assigning weights to points depending on their location in relation to the point to be predicted. When θ = 0, all points are weighted equally, and the S-map model corresponds to an autoregressive model (i.e., a linear model). When θ > 0, nearby points receive greater weights, and the S-map corresponds to a nonlinear model. For coyotes, deer, and pumas, predictability ($\rho$) increased when θ > 0, thereby indicating nonlinear dynamics. At higher values of θ, predictability degrades as the S-map model overfits.


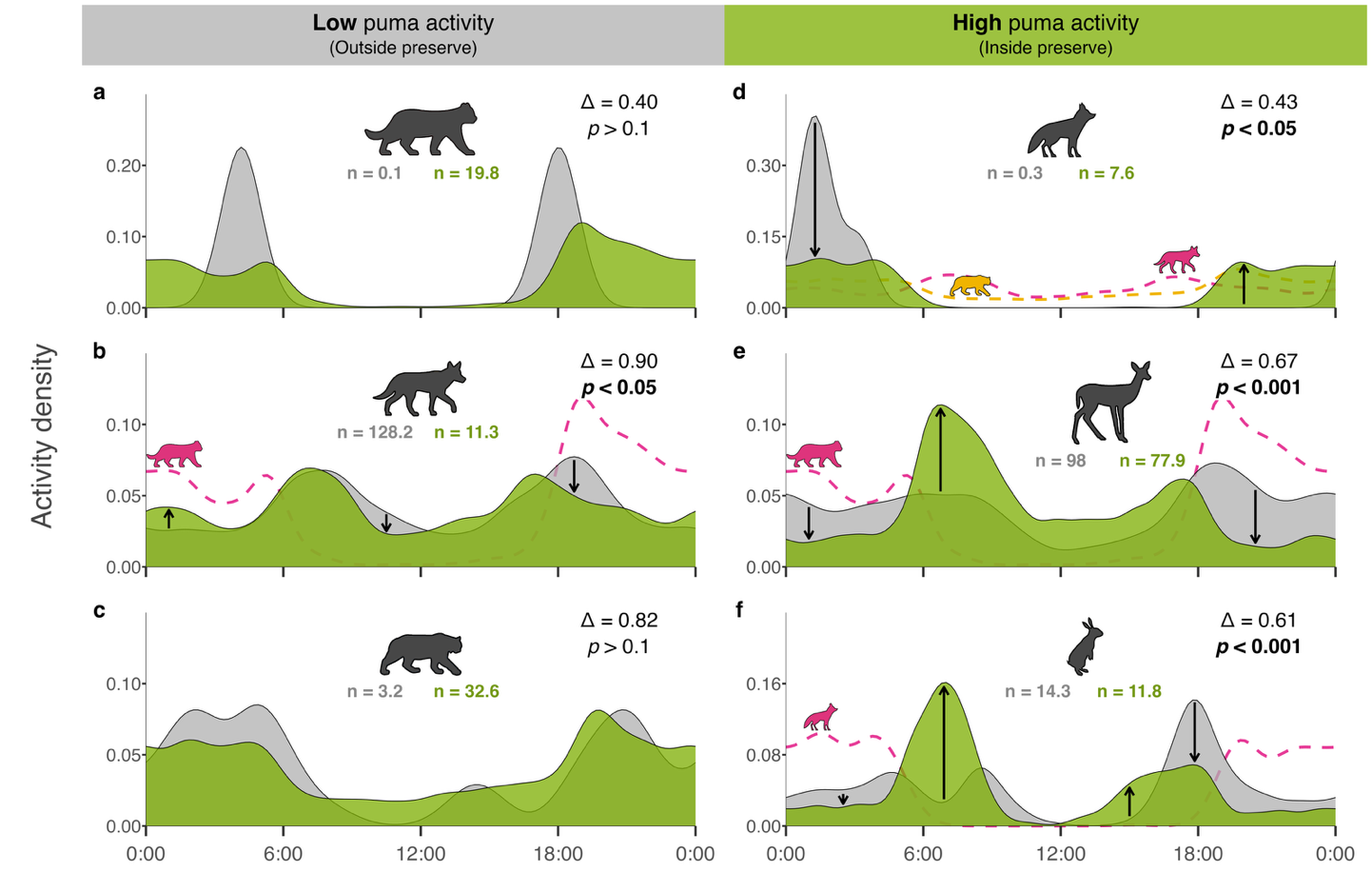


**Fig. S12: Kernel density estimation of daily activity patterns under low and high puma activity. a-f**, Activity patterns of species outside (grey) and inside the preserve (green) during 2019–2022 (low and high puma activity across space). Overlap coefficient (∆) indicates similarity between activity patterns during low and high puma activity for each species (95% CIs in Table S4). *p* value indicates if the two activity patterns were different using the Watson’s U^2^ test (test statistics in Table S4). Where activity patterns are different, arrows indicate how activity patterns changed from low to high puma activity. n indicates the number of independent detections per year per camera for each sample. Activity patterns, under high puma activity, of species that pose predation and intraguild killing threats are displayed in dashed lines.

**Table S3: Summary statistics from comparing daily activity patterns of wildlife across low and high puma activity across time (Fig. 3a–f).** n indicates number of independent detections per year per camera for under low and high puma activity. Overlap indicates overlap coefficient of activity patterns during low and high puma activity for each species, with 95% CIs calculated from 1000 bootstraps. Watson’s U^2^ test and associated *p* value indicate if the activity patterns of the species were different between low and high puma activity.


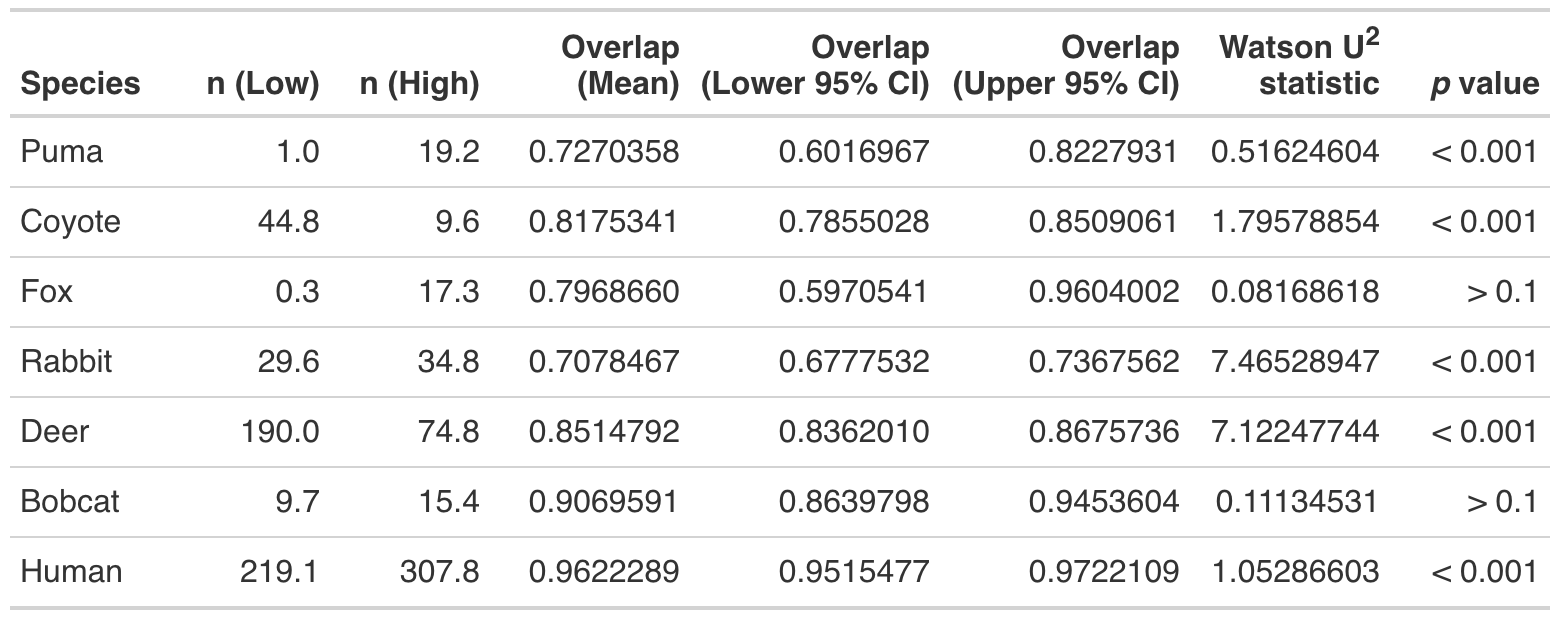


**Table S4: Summary statistics from comparing daily activity patterns of wildlife across low and high puma activity across space (Fig. S12a–f).** n indicates number of independent detections per year per camera for under low and high puma activity. Overlap indicates overlap coefficient of activity patterns during low and high puma activity for each species, with 95% CIs calculated from 1000 bootstraps. Watson’s U^2^ test and associated *p* value indicate if the activity patterns of the species were different between low and high puma activity.


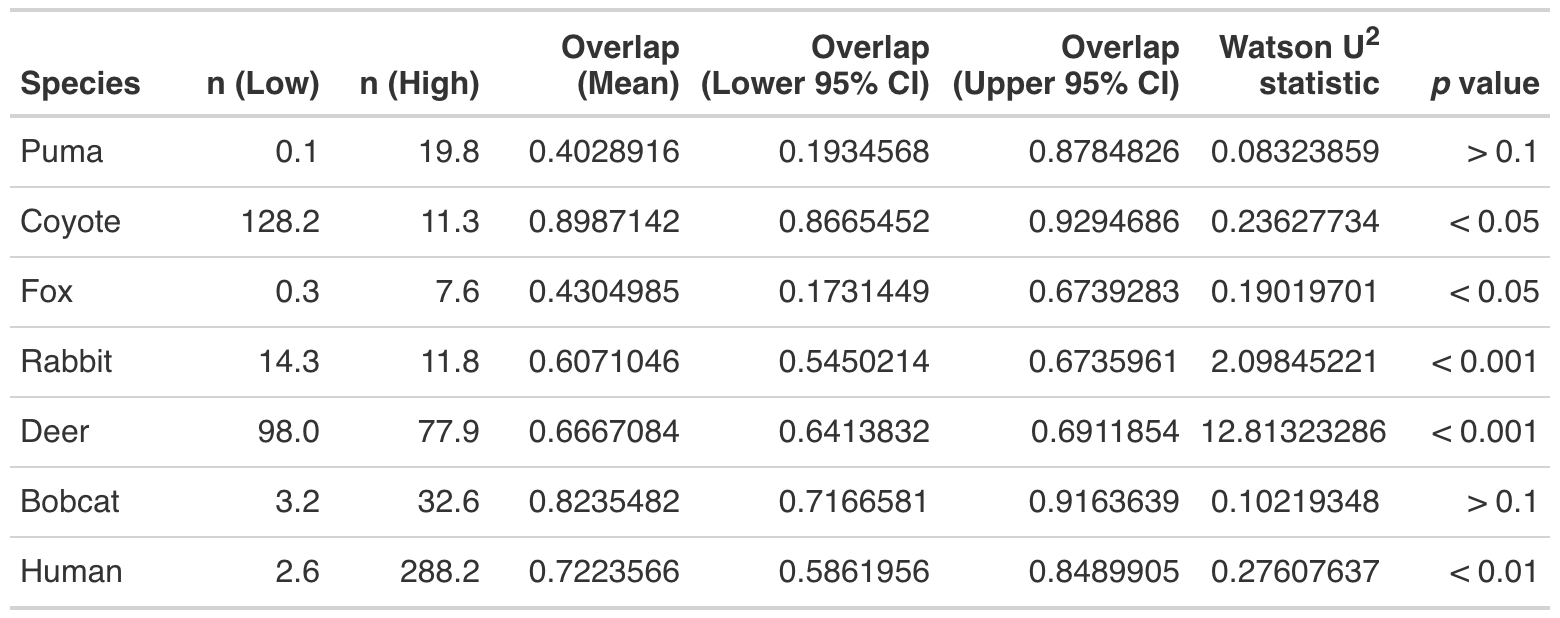


**Table S5: Changes in activity pattern overlap between species across low and high puma activity across time (Fig. 3a–f).** Overlap (without change) indicates the overlap coefficient (with 95% CIs calculated from 1000 bootstraps) between Species A under low puma activity and Species B under high puma activity. Overlap (with change) indicates the overlap coefficient (with 95% CIs calculated from 1000 bootstraps) between Species A and B under high puma activity. Only species which significantly shifted their daily activity patterns are assessed here.


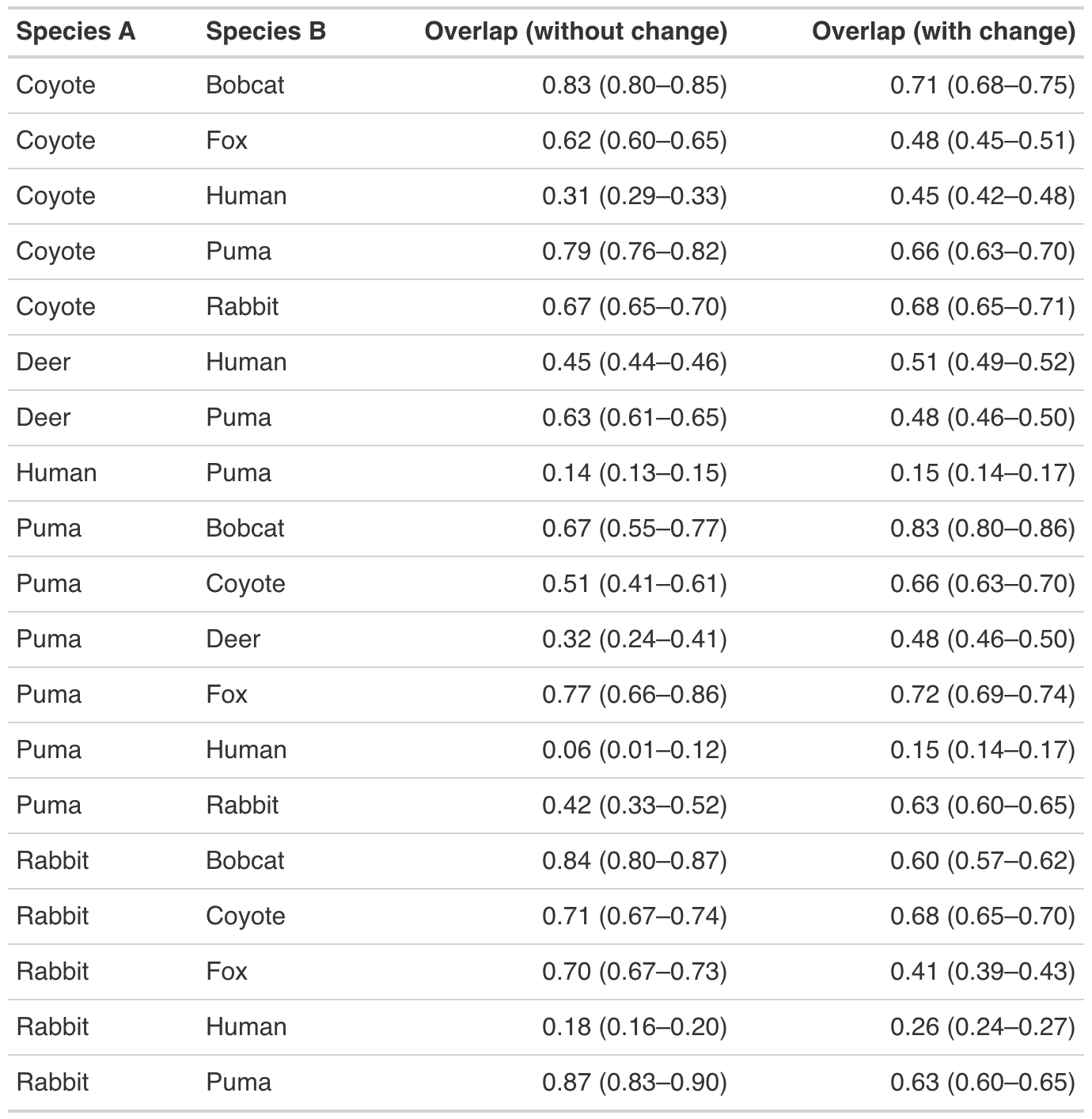


**Table S6: Changes in activity pattern overlap between species across low and high puma activity across space (Fig. S12a–f).** Overlap (without change) indicates the overlap coefficient (with 95% CIs calculated from 1000 bootstraps) between Species A under low puma activity and Species B under high puma activity. Overlap (with change) indicates the overlap coefficient (with 95% CIs calculated from 1000 bootstraps) between Species A and B under high puma activity. Only species which significantly shifted their daily activity patterns are assessed here.


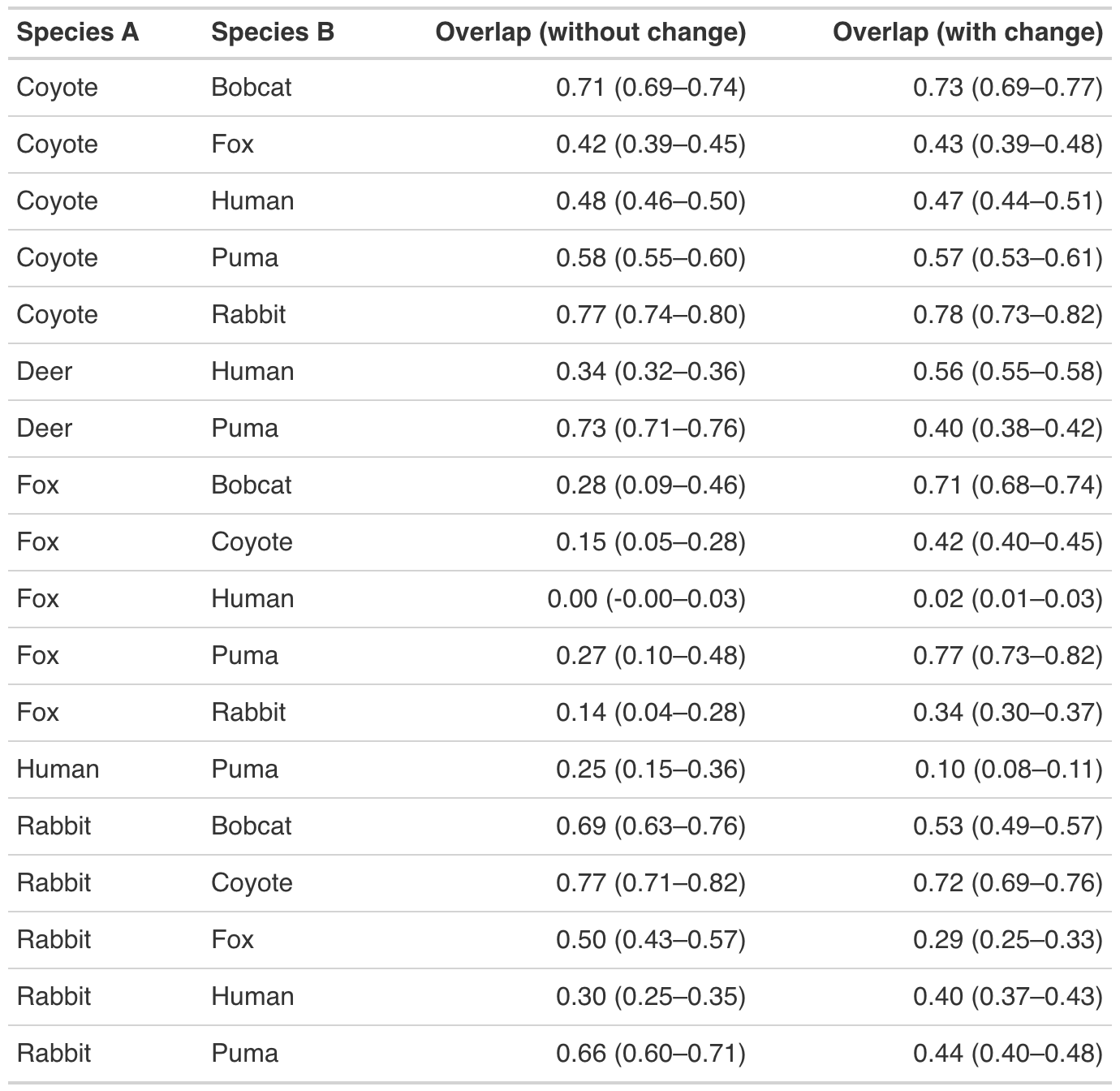


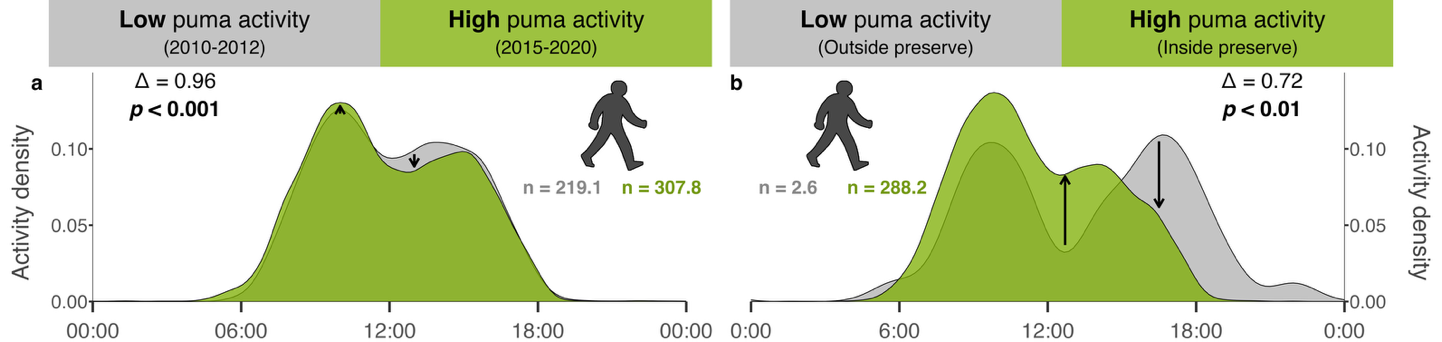


**Fig. S13: Kernel density estimation of human daily activity patterns under low and high puma activity. a**, Activity patterns of humans during November 2010–December 2012 (grey) and January 2015–March 2020 (green) at Jasper Ridge Biological Preserve ('Ootchamin 'Ooyakma). **b**, Activity patterns of humans outside (grey) and inside the preserve (green) during July 2019–December 2022. Overlap coefficient (∆) indicates similarity between activity patterns during low and high puma activity (95% CIs in Tables S3 and S4). *p* value indicates if the two activity patterns were different using the Watson’s U^2^ test (test statistics in Tables S3 and S4). Where activity patterns are different, arrows indicate how activity patterns changed from low to high puma activity. While the Watson’s U^2^ statistics indicated changes, the range of hours for human activity remained similar (predominantly diurnal). n indicates number of independent detections per year per camera for each sample.


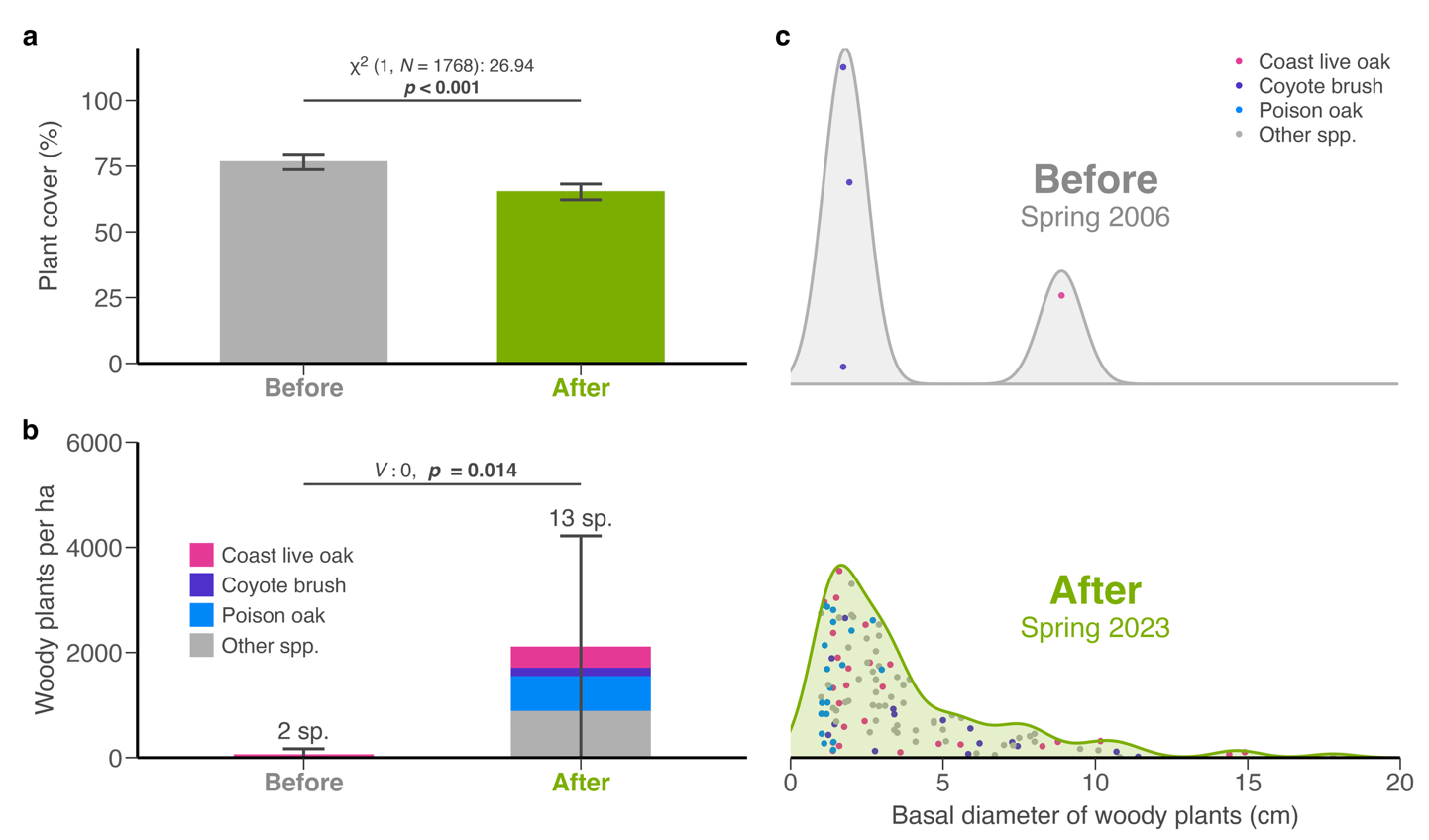


**Fig. S14: Vegetation composition in quadrats sampled in both 2006 and 2023. a**, Plant cover estimated from 10 m × 10 m quadrats, using the line intercept method. Statistically significant difference between years was evaluated using a Chi-squared test. **b**, Woody plant density estimated by counting woody plants with basal diameter ≥ 1 cm in each of the sampled 10 m × 10 m quadrats. Statistically significant difference between years was evaluated using paired Wilcoxon signed-rank test. In a-b, error bars indicate 95% CIs, and Holm–Bonferroni corrections are applied to *p* values. **c**, Distribution of basal diameters of woody plants with basal diameters ≥ 1 cm and ≤ 20 cm. In a-c, grey indicates year with low puma activity (2006), and green indicates year with high puma activity (2023).**Fig. S15: Change in vegetation over time.** Each photograph captures the same location in May of 2012 (**a**), 2016 (**b**), and 2023 (**c**). Density of woody plants increases over time, particularly in the background.


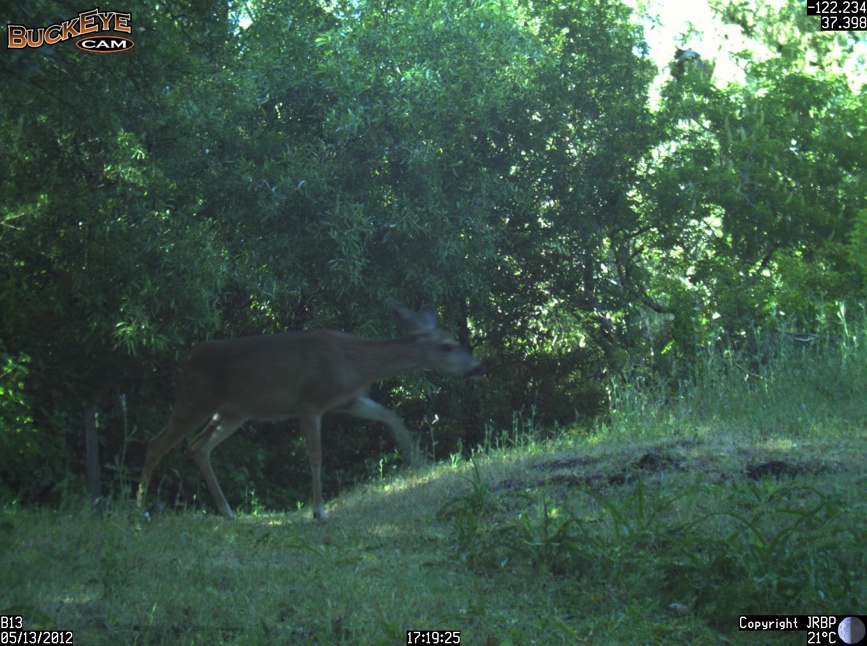

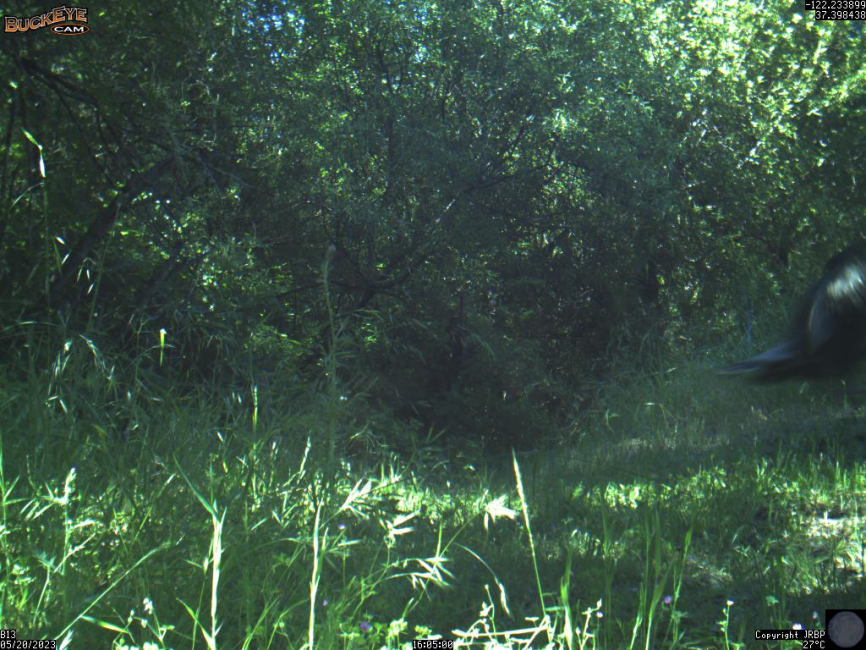

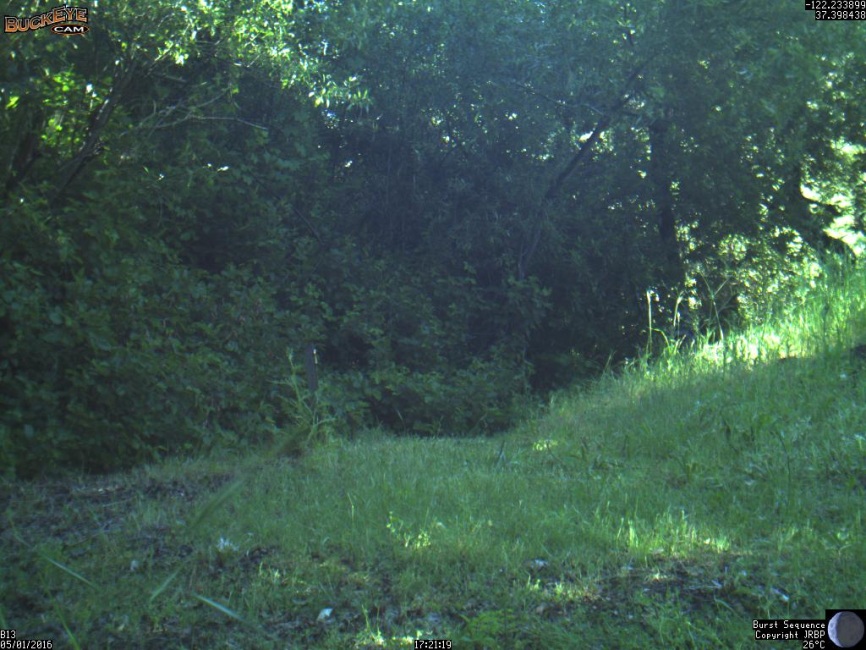


a

b

c

2012

2016

2023


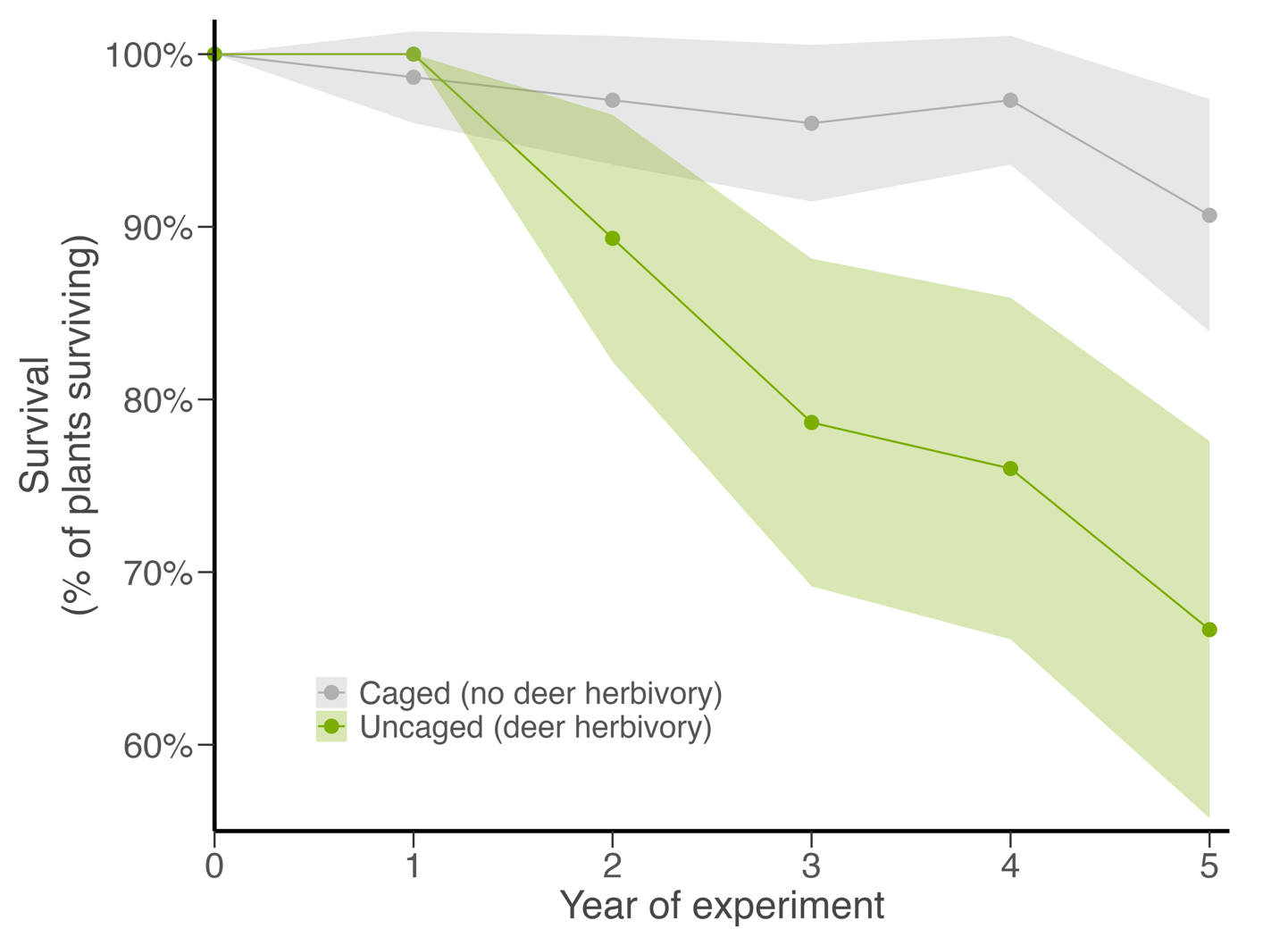


**Fig. S16: Deer herbivory increases mortality of coast live oak (*Quercus agrifolia*) saplings.** In 2009 (Year 0), 75 oak saplings were individually caged at the preserve to exclude deer herbivory and paired with 75 adjacent uncaged saplings (mean pairwise distance = 1.4 m) exposed to herbivory. The matched-pair design allowed for direct comparison of survival over time. Shaded bands represent 95% confidence intervals. Data were provided from a separate, ongoing study at the preserve. Over five years, deer herbivory reduced sapling survival by 26%, providing causal evidence that deer can suppress woody plant recruitment.

**Table S7: Precipitation and temperature data for each year that vegetation was sampled.** Data are sourced from the National Oceanic and Atmospheric Administration.

| **Year**  (Date of last survey) | **2006**  (8 June 2006) | **2015**  (12 April 2015) | **2023**  (20 May 2023) |
| --- | --- | --- | --- |
| **Rainfall over past one year**  (# days with missing data) | 912.8 mm (11 days) | 398.2 mm (15 days) | 1053.4 mm (4 days) |
| **Rainfall over past two years** (# days with missing data) | 1834.6 mm (56 days) | 864.3 mm (52 days) | 1462.1 mm (7 days) |
| Rainfall data station | USC00049792  Woodside Fire Station 1, CA, USA | US1CASM0022  Woodside 3.4 S, CA, US | US1CASM0022  Woodside 3.4 S, CA, US |
| **Mean temperature** (°C) over past one year | 13.0 °C | 14.4 °C | 12.6 °C |
| **5^th^ and 95^th^ percentile of temperatures** (°C) | 7.2 – 18.7 °C | 8.9 – 20.1 °C | 6.1 – 19.8 °C |
| Temperature data station | USR0000CLAH  La Honda, CA US | USR0000CLAH  La Honda, CA US | USR0000CLAH  La Honda, CA US |


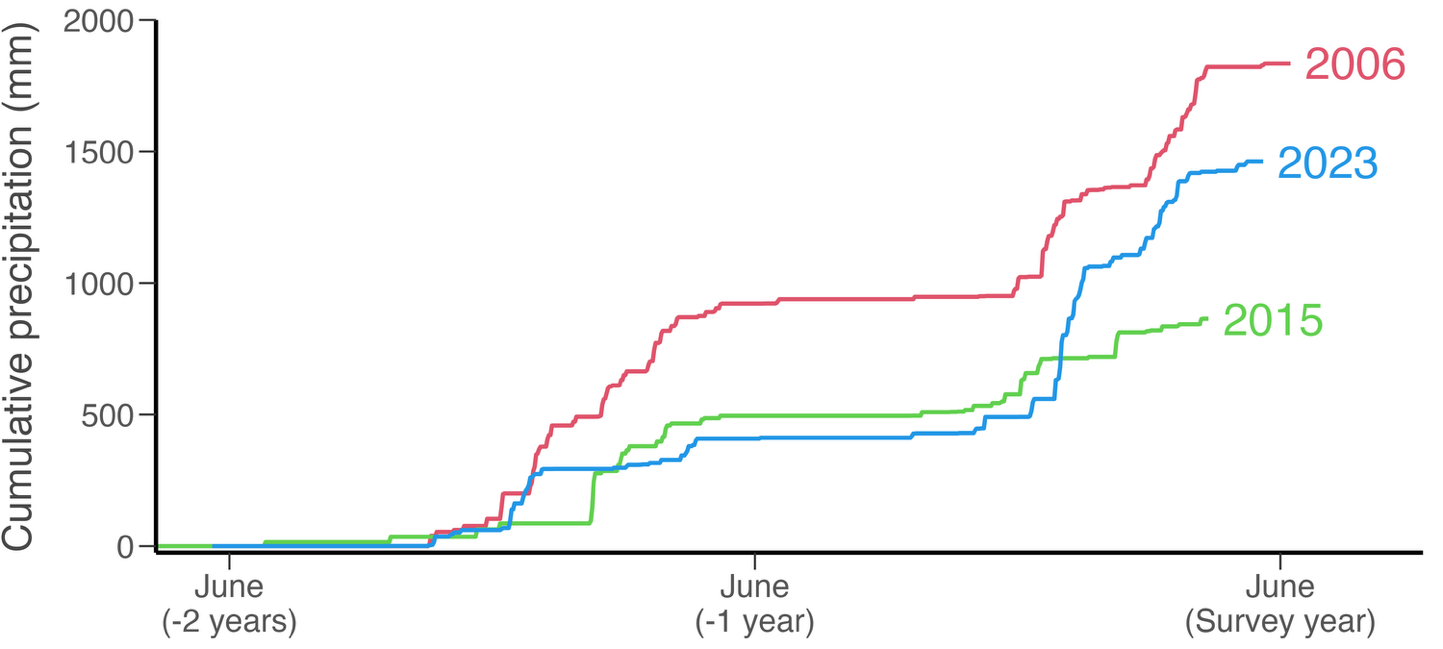


**Fig. S17: Cumulative precipitation over past two years of each vegetation survey.** Data are sourced from the National Oceanic and Atmospheric Administration and are based on weather stations in Woodside, California.


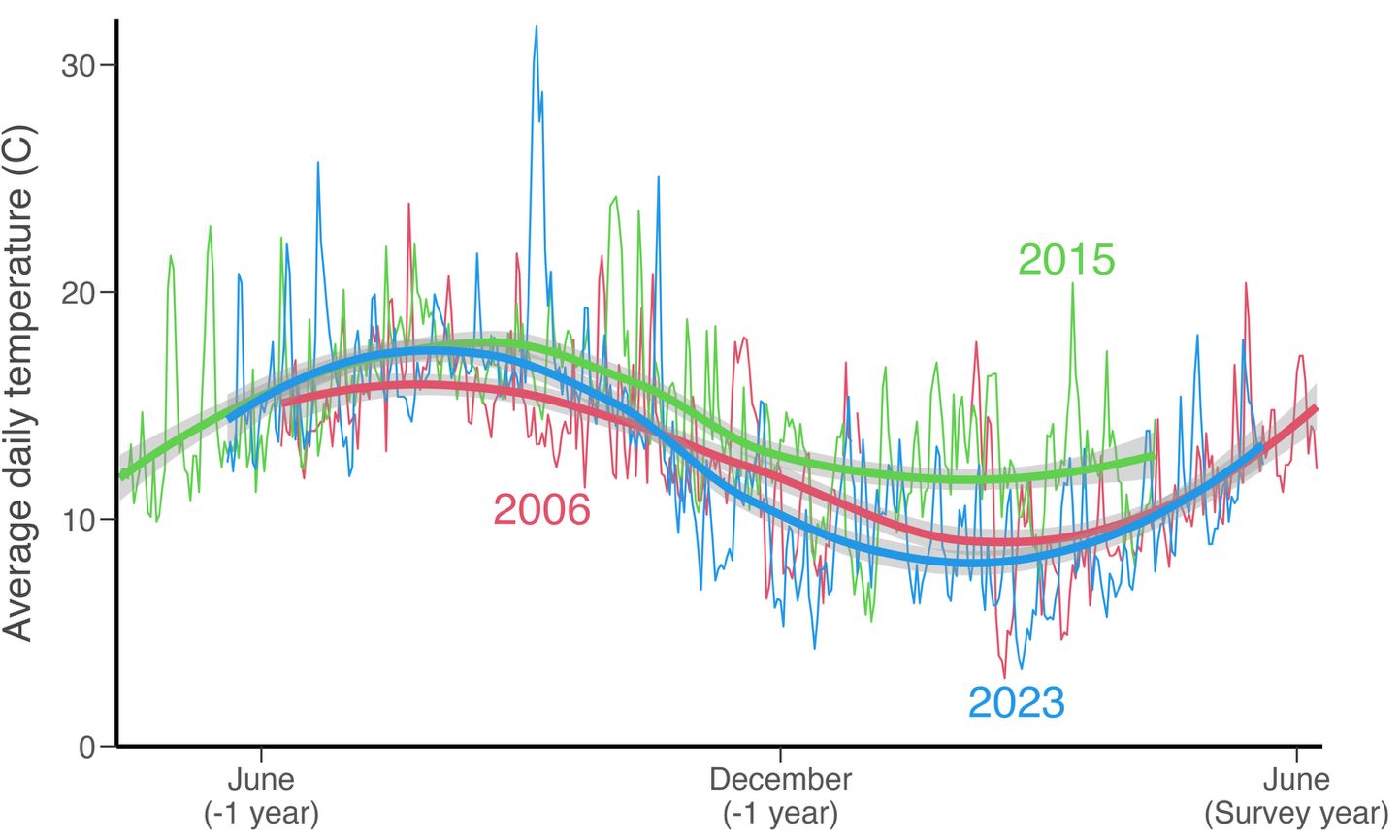


**Fig. S18: Average daily temperature over past one year of each vegetation survey.** Data (thin lines) are sourced from the National Oceanic and Atmospheric Administration and are based on weather stations in La Honda, California. Thick lines are loess curves, with grey shading indicating 95% CIs.


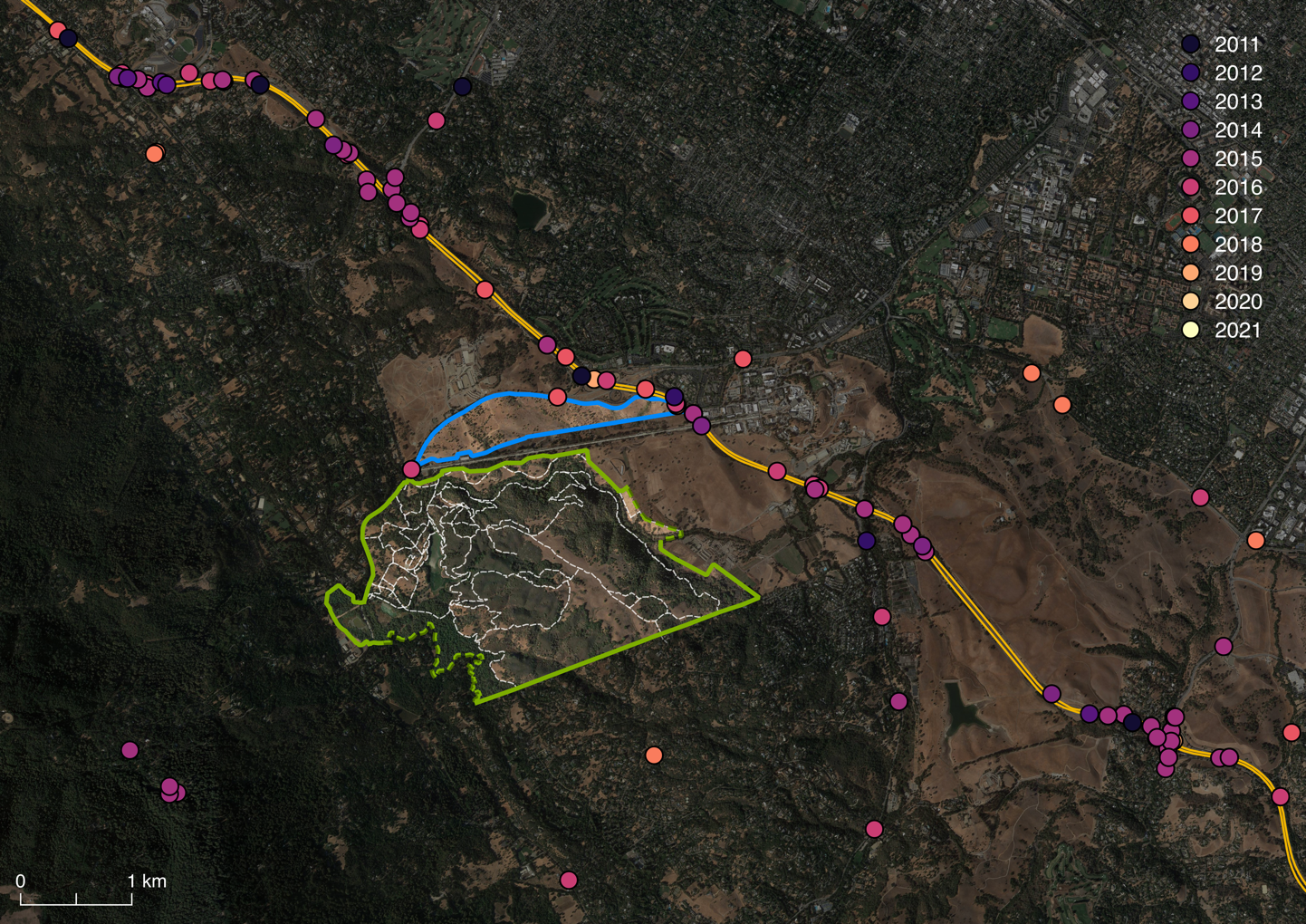


**Fig. S19: Black-tailed deer carcasses recorded on California Roadkill Observation System.** Each point indicates an observation of roadkill recorded by citizen scientists or the California Highway Patrol (Waetjen & Shilling, 2017). Green and blue lines outline the boundaries of the preserve and adjacent monitored area respectively, with solid lines indicating fenced boundaries. White dashed lines indicate trails. Yellow lines indicate the Interstate 280 highway.

**References**

Meta. (2020). *United States: High Resolution Population Density Maps + Demographic Estimates* (population_usa28_-120.tif.zipGeoTIFF) [dataset]. Humanitarian Data Exchange. https://data.humdata.org/dataset/eec3a01f-5237-4896-8059-a6be193ca964/resource/58b480a3-afbd-4d1e-bd9f-5ef480bffb61/download/population_usa28_-120_2019-07-01.tif.zip

Waetjen, D. P., & Shilling, F. M. (2017). Large Extent Volunteer Roadkill and Wildlife Observation Systems as Sources of Reliable Data. *Frontiers in Ecology and Evolution*, *5*. https://www.frontiersin.org/articles/10.3389/fevo.2017.00089
